# Synergistic Action of Phage and Antibiotics: Parameters to Enhance the Killing Efficacy Against Mono and Dual-Species Biofilms

**DOI:** 10.3390/antibiotics8030103

**Published:** 2019-07-25

**Authors:** Ergun Akturk, Hugo Oliveira, Sílvio B. Santos, Susana Costa, Suleyman Kuyumcu, Luís D. R. Melo, Joana Azeredo

**Affiliations:** 1 LIBRO-Laboratório de Investigação em Biofilmes Rosário Oliveira, Centre of Biological Engineering, University of Minho, Campus de Gualtar, 4700-057 Braga, Portugal; 2 Department of Medical Genetics, Medical Faculty, Sifa University, 35535 Izmir, Turkey

**Keywords:** *Pseudomonas aeruginosa*, *Staphylococcus aureus*, bacteriophage, dual-species, biofilms, antibiotic, synergy, simultaneous, sequential

## Abstract

*Pseudomonas aeruginosa* and *Staphylococcus aureus* are opportunistic pathogens and are commonly found in polymicrobial biofilm-associated diseases, namely chronic wounds. Their co-existence in a biofilm contributes to an increased tolerance of the biofilm to antibiotics. Combined treatments of bacteriophages and antibiotics have shown a promising antibiofilm activity, due to the profound differences in their mechanisms of action. In this study, 48 h old mono and dual-species biofilms were treated with a newly isolated *P. aeruginosa* infecting phage (EPA1) and seven different antibiotics (gentamicin, kanamycin, tetracycline, chloramphenicol, erythromycin, ciprofloxacin, and meropenem), alone and in simultaneous or sequential combinations. The therapeutic efficacy of the tested antimicrobials was determined. Phage or antibiotics alone had a modest effect in reducing biofilm bacteria. However, when applied simultaneously, a profound improvement in the killing effect was observed. Moreover, an impressive biofilm reduction (below the detection limit) was observed when gentamicin or ciprofloxacin were added sequentially after 6 h of phage treatment. The effect observed does not depend on the type of antibiotic but is influenced by its concentration. Moreover, in dual-species biofilms it was necessary to increase gentamicin concentration to obtain a similar killing effect as occurs in mono-species. Overall, combining phages with antibiotics can be synergistic in reducing the bacterial density in biofilms. However, the concentration of antibiotic and the time of antibiotic application are essential factors that need to be considered in the combined treatments.

## 1. Introduction

Polymicrobial interactions are widespread in many biofilm-associated infections [1], accounting for a significant higher mortality and considerable high costs to the health-care systems [2,3]. Biofilms are communities of microbial cells adhered to biotic or abiotic surfaces and encased in a self-produced extracellular polymeric matrix that confer protection to the community against adverse environmental conditions and antimicrobials including the presence of antibiotics [4]. In an established polymicrobial biofilm, the co-existence of different species is a clear advantage for the overall biofilm population. These biofilms very often exhibit improved capabilities and functions compared to single-species ones [5,6], such as enhanced degradation of organic compounds [7], increased virulence [8], and increased tolerance against antimicrobials [9].

*Pseudomonas aeruginosa* and *Staphylococcus aureus* are versatile bacterial pathogens and common etiological agents of polymicrobial associated infections. These two opportunistic pathogens exhibit intrinsic and acquired antibiotic resistance [10]. When co-existing in a biofilm this tolerance largely increases, namely due to the decreased metabolic activity, increased bacterial doubling time, and increased level of mutations and upregulation of efflux pumps [11].

Bacteriophages (or phages), viruses that infect bacteria, are natural antibacterial agents that specifically infect and lyse bacteria. Phages are the most abundant biological entities on our planet and can be used as biocontrol agents targeting bacterial cells either in suspension or in biofilms [12,13]. Due to the phage bacterial host specificity and bacteriolytic activity against antibiotic-resistant strains, phage therapy has been suggested as a valuable approach to control numerous pathogenic bacteria. Phages can penetrate the inner layers of the biofilms and infect dormant cells [14], which is a clear advantage of phages compared to antibiotics in killing biofilms. Therefore, it has been proposed that phages may be a useful combination with antibiotic treatment [15,16,17]. Several studies demonstrated the efficiency of phage and antibiotic combinations in planktonic cultures of *P. aeruginosa* [17,18] and biofilms [19]. Besides, phages and antibiotics use different mechanisms of action, which make them effective against phage/antibiotic resistance pathogens [17]. Consequently, antibiotic and phage resistance have a low chance of evolving at the same time and, besides, bacteria resistant to one agent will be taken by the other agent. However, to our knowledge, this type of studies was never assessed on dual-species biofilms.

In this study, we report the isolation and characterization of a new *Pakpunavirus* phage, named vB_PaM_EPA1, and the use of several phage-antibiotic combinations against mono and dual-species biofilms of *P. aeruginosa* and *S. aureus*.

## 2. Results

### 2.1. Isolation and Characterization of a New P. aeruginosa-Infecting Phage

**Figure 1:**
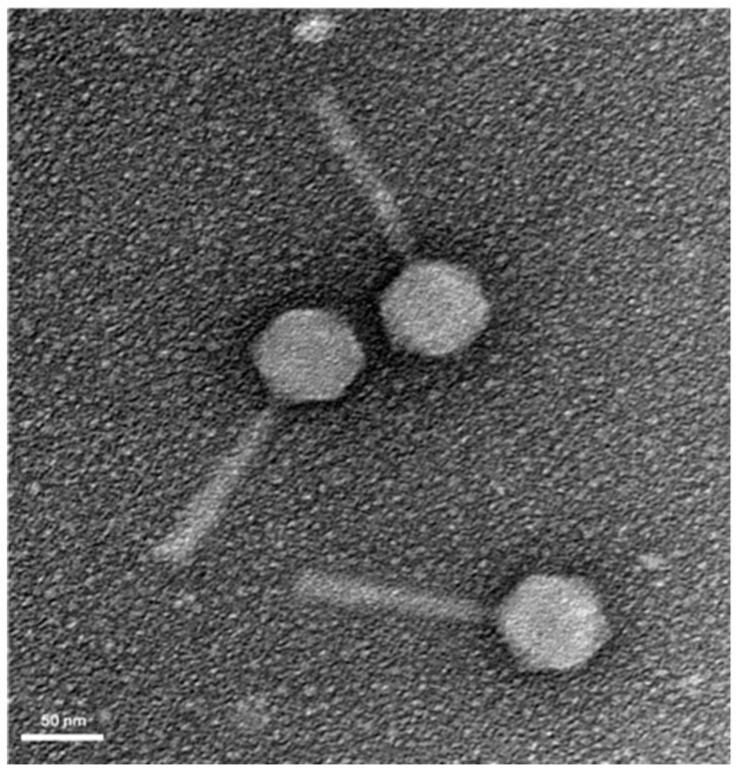
TEM image of *P. aeruginosa* specific phage EPA1 obtained by negative staining with 2% (*w/v*) uranyl acetate. Scale bar represents 50 nm.

Six clinical strains were used for phage enrichment (Table S1), using raw sewage from Sifa Hospital (Izmir, Turkey) as phage sources. A phage vB_PaM_EPA1 (EPA1) was isolated, and its plaque morphology was characterized by clear and small plaques (0.8 mm in diameter) surrounded by halo rings on the host strain Sifa_Pa_1.5 (Table S1). The morphology of EPA1 particles was observed by Transmission Electron Microscopy (TEM). EPA1 has an icosahedral head with 69 nm in diameter and a contractile tail of 145 × 24 nm. According to Ackermann’s classification [20], EPA1 belongs to the *Caudovirales* order and *Myoviridae* family (Figure 1).

### 2.2. Host range, Efficiency of Plating and One-Step Growth Curve

In total, seventeen drug-resistant clinical isolates (Table S1) and three reference *P. aeruginosa* strains were used to determine the host range and the efficiency of plating (EOP) of EPA1 (Table S1). EPA1 has a broad spectrum of activity (within the panel of strains used) and was able to propagate on 70% (14 out of 20) of the *P. aeruginosa* strains with moderate to high EOP. No lysis from without events were observed. Also, no correlation between phage susceptibility and antibiotic resistance was detected (Table S1). Due to the fact that EPA1 propagates better in *P. aeruginosa* PAO1 strain, we have used this strain to produce the phage for further experiments. Nevertheless, we are aware of the fact that PAO1 encloses filamentous phages that could influence phage production, however no filamentous phages were detected by plating methods or TEM [21]. One-step growth curve (OSGC) experiments were performed to examine the infection parameters of EPA1. The latent period of EPA1 was around 10 min, and the burst size was approximately of 34 progeny phages per infected cell (Figure S1). 

### 2.3. Genome Analysis of EPA1

EPA1 has a linear double-stranded DNA genome containing 91,394 bp with an average 49.2% GC content. This phage encodes 175 putative CDSs, of which 35 have a putative function, and 140 are considered hypothetical/novel (Table S2). Most predicted gene products exhibit homology to phage known proteins belonging to the *Pakpunavirus* (ICTV 2015.029a-dB ratification) genus, mostly *Pseudomonas* phages JG004 (NC_019450.1), PAK_P4 (NC_022986) and vB_PaeM_C2-10_Ab1 (NC_019918). Moreover, seventeen tRNA genes coding for Arg, Asn, Asp, Cys, Gln, Glu, Gly, Ile, Lys, Leu, Met, Phe, Pro, Ser, Thr, Trp and Tyr were found. Regarding regulatory elements, 16 promoters were identified as well as 14 rho-independent terminators. The general characteristics of the phage genome are summarized in Table 1. Whole-genome comparisons through BLASTN show that EPA1 has a high overall nucleotide identity (>90%) with other *P. aeruginosa* phages, such as JG004 (NC_019450.1), vB_PaeM_SCUT-S2 (MK340761.1) and SRT6 (MH370478.1). EPA1 shares >145 proteins with these phages.

### 2.4. Characterisation of Mono and Dual-Species Biofilm Models

In vitro mono and dual-species biofilms were formed in 24-well polystyrene plates for 48 h, and the number of viable bacteria cells were determined by colony forming unit (CFU) counting. It is well documented that *P. aeruginosa* inhibits *S. aureus* proliferation in dual-species biofilms. The reason for the lower density of *S. aureus* population has been attributed to the toxic effect of *P. aeruginosa* exoproducts [22], including LasA protease, 4-hydroxy-2-heptylquinoline-N-oxide (HQNO) [23], the Pel and Psl products [24], and phenazines such as pyocyanin [25]. Therefore, in order to successfully produce dual-species biofilms, biofilm formation has been initiated with an *S. aureus* cell culture, and 24 h later *P. aeruginosa* cells were added on the *S. aureus* biofilm, then incubated for another 24 h. A similar strategy of biofilm formation was also used by DeLeon et al. [26] where biofilms were initiated with *S. aureus,* and 48 h later *P. aeruginosa* cells were added [26]. Our results showed that the number of viable cells of *S. aureus* was 3.77 × 10^7^ CFU/mL and 1.2 × 10^9^ CFU/mL for *P. aeruginosa* in the mono-species biofilms. Regarding the dual-species biofilms, the concentrations were 1.28 × 10^7^ CFU/mL for *S. aureus* and 2 × 10^8^ CFU/mL for *P. aeruginosa*.

### 2.5. Biofilm Treatments

The selected antibiotics (Table 2) and EPA1 were tested individually or in combinations within intact mono, and dual-species 48 h biofilms and treated for 24 h in total. Phage and antibiotics were simultaneously or sequentially added in combined treatments. Twenty-four hours post-treatment, CFUs were enumerated in order to assess the antibiofilm efficacy and to characterize the possible interactions between antimicrobials.

Further, the effects of phage, gentamicin at MIC and phage-gentamicin at MIC combinations (simultaneous and sequential) in mono and dual-species biofilms were also analyzed by confocal laser microscopy (CLSM). For that assessment, fluorescence probes were designed to specifically target differentially both bacterial species. Generally, microscopy analysis corroborated cell counting results.

#### 2.5.1. Antibiotics and Phages Alone cause a Moderate Killing Effect on Biofilms

**Figure 2:**
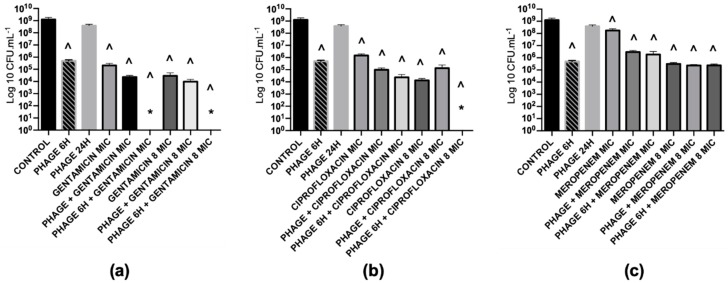
Treatment of *P. aeruginosa* PAO1 48 h biofilms with different antimicrobial agents individually or in combinations; phage EPA1 and (**a**) gentamicin; (**b**) ciprofloxacin; and (**c**) meropenem for 24 h. A prefix PHAGE indicates EPA1 in MOI 1, 6 H and 24 Hindicates treatment time period for 6 and 24 h, MIC indicates the dose of antibiotics with 1-time MIC value of *P. aeruginosa* 8× MIC indicates the dose of antibiotics with 8-times MIC value of *P. aeruginosa*, PHAGE + antibiotic indicates simultaneous treatment, and PHAGE 6 H+ antibiotics indicates phage was added first then antibiotic was added with 6 h delay. * Under detection limit (<10^2^). (**^**) Statistical differences between the control and treated biofilms were determined by two-way repeated-measures analysis of variance (ANOVA) with a Tukey’s multiple comparison test.

Three antibiotics were selected (gentamicin, ciprofloxacin and meropenem), and their anti-biofilm ability was tested against *P. aeruginosa* and *S. aureus* mono-species biofilms. These antibiotics were selected depending on their mechanism of action (Table 2): protein synthesis inhibitor (gentamicin), DNA synthesis inhibitor (ciprofloxacin) and cell wall synthesis inhibitor (meropenem). The killing effect of the antibiotics against *P. aeruginosa* biofilms, used in different concentrations, ranged from 0.8 to 5 orders-of-magnitude (Figure 2).

Regarding *S. aureus,* no significant reduction in the number of viable cells was observed when antibiotics were applied at their MIC (Figure S2). However, when gentamicin was applied with 8xMIC, the number of viable cells was reduced approximately 1.4 orders-of-magnitude (Figure S2).

**Figure 3:**
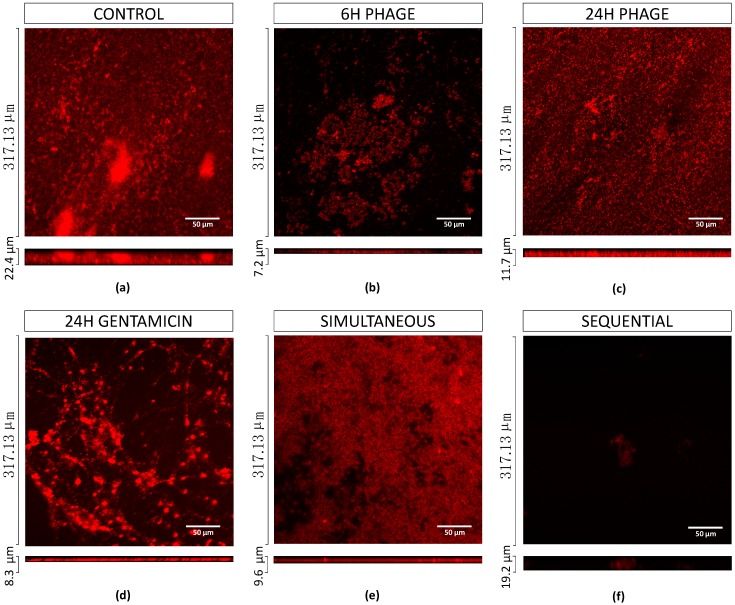
3D reconstructions of confocal stacks of images of mono-species *P. aeruginosa* biofilms. (**a**) Control, (**b**) 6 h phage treatment, (**c**) 24 h phage treatment, (**d**) 24 h Gentamicin treatment, (**e**) 24 h simultaneous treatment, (**f**) 24 h sequential treatment. All biofilms were stained with EPA1_TFP (with mCherry) recombinant protein. Scale bar represents 50 μm.

Additionally, EPA1 was individually tested (at multiplicity of infection, MOI, of 1) on *P. aeruginosa* biofilms for 6 h and 24 h. The observed reductions were 3.4 and 0.5 orders-of-magnitude, respectively (Figure 2, Figure 3b,c). The best reduction was observed at 6 h post-treatment; after that, *P. aeruginosa* cells started to regrow (Figure 2). CLSM images corroborated CFUs results. *P. aeruginosa* biofilms after being challenged for 6 h with EPA1 reduced their thickness from 22.4 μm to 7.2 μm, but after 24 h of phage contact an increase in biofilm thickness to 11.7 μm was observed (Figure 3a–c).

#### 2.5.2. Combined Treatments with Simultaneous Application of Phage and Antibiotics have Synergistic Effects for Low Concentrations of Antibiotics

The efficacy of the combinations of phage and antibiotics was also tested on *P. aeruginosa* mono-species biofilms. These maturated intact biofilms were treated in one of two ways; simultaneously (phage and antibiotic were added at the same time) and sequentially (phage was added first, then antibiotic was added with a delay of 6 h).

The combination of phage EPA1 with gentamicin (Figure 3e), ciprofloxacin, or meropenem when applied simultaneously with a lower dose (MIC) resulted in population reductions of 4.7, 4.1 and 2.6 orders-of-magnitudes (Figure 2), respectively. These results show a clear synergistic effect between antimicrobial agents in most cases (Table 3). When the antibiotic concentrations were increased, we were expecting an increase in the killing efficacy of simultaneous combined treatments, however, interestingly 8 × MIC did not increased the overall biofilm killing (in certain cases, we observed an antagonistic effect) (Table 3).

#### 2.5.3. Antibiotics that Target Protein and DNA Synthesis Mechanisms Interfere with Phage Replication

In order to understand why increasing the antibiotic concentration did not lead to an increased killing activity, we tested the effect of the antibiotics on phage replication. Phage titer was enumerated after 24 h of simultaneous treatment and compared with the control. Unsurprisingly, the titer of phages, when combined with gentamicin and ciprofloxacin was significantly lower than the titer of phages in control samples (Figure S3). Conversely and as expected, the phage replication was not affected by the presence of meropenem. This antibiotic is affecting bacteria cell wall synthesis and thus does not interfere in phage replication.

#### 2.5.4. Combined Treatments with Sequential Application of Phage and Antibiotics have a better Killing Efficacy than when Applied Simultaneously

The fact that protein and DNA synthesis inhibitors interfere with phage replication, led us to assess a sequential treatment in which the phage was applied first and six hours later the antibiotic. This six hour period was chosen based on previous biofilm/phage interaction studies that refer that after six hours of phage interaction, a regrowth of phage-resistant phenotypes is observed [27]. Our CFU and CLSM results have also corroborated this phenomenon (Figure 2a and Figure 3b,c).

The same phage-drug combinations tested before were applied in sequential treatments. The results showed an almost eradication of the biofilm with gentamicin (MIC, 8× MIC) (Figure 3f) and ciprofloxacin (8xMIC). Besides, other combinations with ciprofloxacin or meropenem (with MIC) also showed an increased killing effect, 4.7 and 2.8 orders-of-magnitudes, respectively. In accordance, increasing the antibiotic concentration of meropenem in sequential treatment (to 8× MIC), resulted in an antagonistic effect (3.7 orders-of-magnitudes), contrarily to what was observed for the other antibiotics (Figure 2). CLSM results also confirmed that almost all biofilm was eradicated except a cluster (Figure 3e). To understand the impact of antimicrobial application order in sequential interaction, the same combinations were applied in the reverse order. Gentamicin was applied first, and then phage was applied six hours after. The collected data showed that killing efficacies of combinations were reduced when gentamicin MIC and 8× MIC were applied first, with reductions of 2.5 and 3.6 orders-of-magnitude, respectively (Figure S4). 

The data suggest that biofilm exposure to phage prior to antibiotics is more effective than simultaneous treatment in eliminating biofilm-associated cells. Considering the overall results, when gentamicin was administered at MIC sequentially after six hours of phage addition, it almost eradicated biofilms (Figure 3f). 

#### 2.5.5. The Phage Killing Efficacy with the Sequential Treatment of Phage and Gentamicin cannot be Extrapolated to other Protein Synthesis Inhibitors

**Figure 4:**
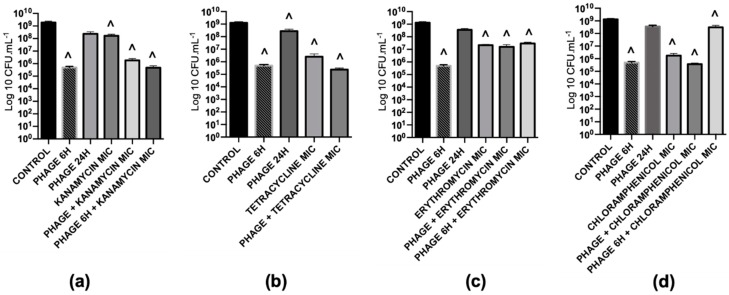
Treatment of *P. aeruginosa* PAO1 48 h biofilms with protein synthesis inhibitor antimicrobial agents individually or in combinations for 24 h. (**a**) Kanamycin is a 30S protein synthesis inhibitor; (**b**) Tetracycline is a 30S protein synthesis inhibitor; (**c**) Erythromycin is a 50S protein synthesis inhibitor; (**d**) Chloramphenicol is a 50Ss protein synthesis inhibitor. A prefix PHAGE indicates EPA1 in MOI 1, 6 H and 24 H indicates treatment time period for 6 and 24 h, MIC indicates the dose of antibiotics with 1-time MIC value of *P. aeruginosa*, PHAGE + antibiotic indicates simultaneous treatment and PHAGE 6 H + antibiotics indicates that phage was added first, then antibiotic was added with 6 h delay. (**^**) Statistical differences between the control and treated biofilms were determined by two-way repeated-measures analysis of variance (ANOVA) with a Tukey’s multiple comparison test.

An impressive biofilm biomass reduction was observed with a protein synthesis inhibitor (gentamicin) at MIC. To understand if this effect can be extrapolated to other antibiotics of the same class, we also tested kanamycin, tetracycline, erythromycin and chloramphenicol (Table 3), in simultaneous and sequential combinations. Contrarily to what we were expecting, the effect observed for gentamicin was not reproduced with the other tested antibiotics (Figure 4). In fact, those antibiotics alone had a low to moderate effect against biofilms, lower than 3 orders-of-magnitude in the overall biomass reduction. Gentamicin alone caused ten times more biomass damage, which could be one of the reasons for the better performance of sequential treatments with gentamicin compared to the other antibiotics.

#### 2.5.6. The Efficacy of Sequential Antibiofilm Treatments is Dependent on the Antibiotic Concentration

**Figure 5:**
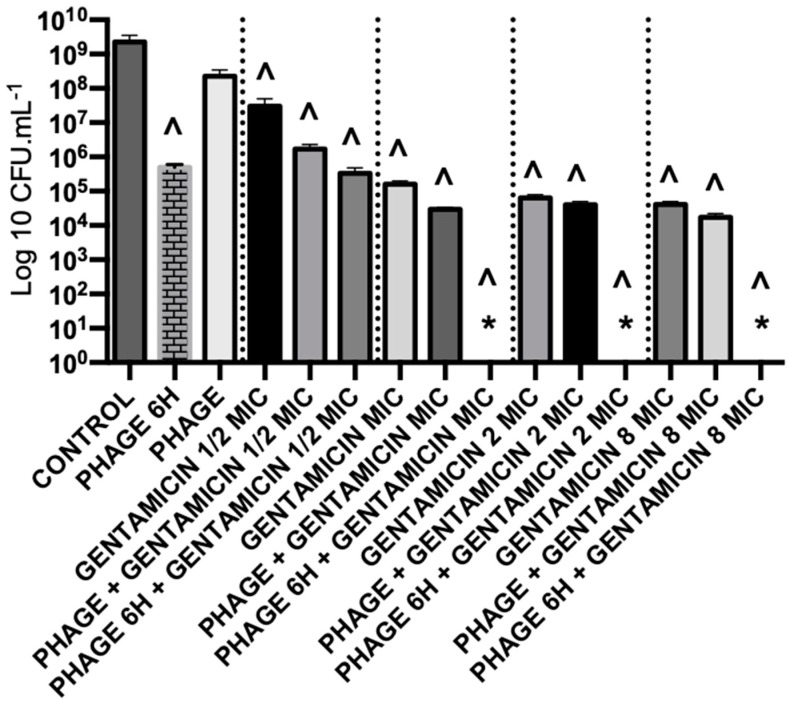
Treatment of *P. aeruginosa* PAO1 48 h biofilms with gentamicin at different concentrations. A prefix PHAGE indicates EPA1 in MOI 1, 1/2 MIC indicates the dose of antibiotics with 1/2× MIC value, MIC indicates the dose of antibiotics with 1× MIC value of *P. aeruginosa*, 2 MIC indicates the dose of antibiotics with 2× MIC value of *P. aeruginosa*, 8 MIC indicates the dose of antibiotics with 8× MIC value of *P. aeruginosa*, PHAGE + antibiotic indicates simultaneous treatment and PHAGE 6 H + antibiotics indicates phage was added first then antibiotic was added with 6 h delay. * Under detection limit (<10^2^). (**^**) Statistical differences between the control and treated biofilms were determined by two-way repeated-measures analysis of variance (ANOVA) with a Tukey’s multiple comparison test.

We also investigated the effect of the different gentamicin concentrations on the simultaneous and sequential treatment efficacy (Figure 5). A direct correlation was observed between the concentration of gentamicin and the biofilm killing efficacy. An almost complete biofilm eradication (below the detection limits) was observed only when antibiotic concentrations were equal or above the MIC (Table S1).

#### 2.5.7. Sequential Application of Phages and Gentamicin have a great Antibiofilm Effect in Dual-Species Biofilms

**Figure 6:**
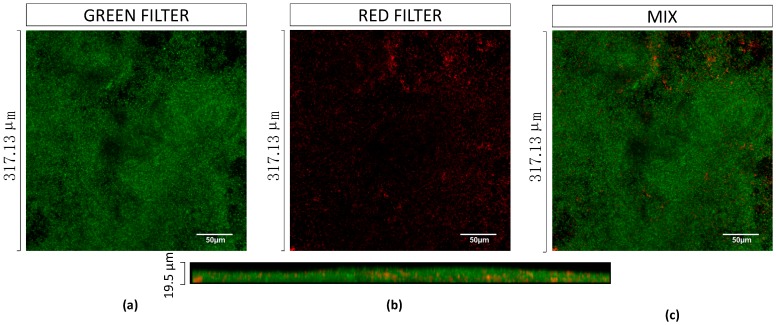
3D reconstructions of confocal stacks of images of dual-species of *P. aeruginosa* and *S. aureus* biofilms. (**a**) 48 h old intact biofilms were stained by using recombinant proteins, LM12_AMI-SH3 (with GFP) specific for *S. aureus* and (**b**)EPA1_TFP (with mCherry) specific for *P. aeruginosa*. (**c**) 48 h old intact biofilms were stained by using both recombinant proteins. Scale bar represents 50 μm.

**Figure 7:**
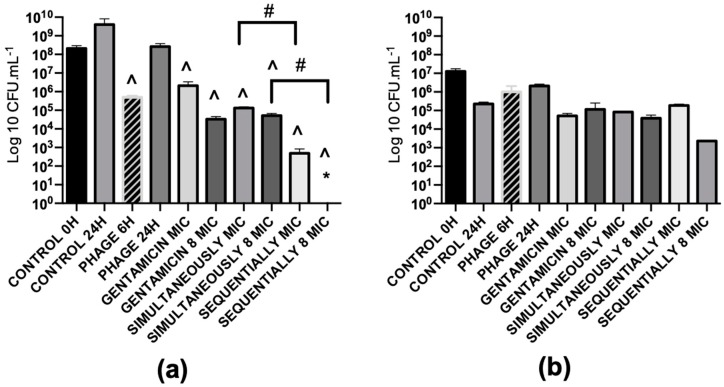
Treatment of 48 h dual-species biofilm. (**a)** *P. aeruginosa* number of viable cells. (**b)** *S. aureus* number of viable cells. A prefix PHAGE indicates EPA1 in MOI 1, 6 H and 24 H indicates treatment time period for 6 and 24 h, MIC indicates the dose of antibiotics with 1× MIC value of *P. aeruginosa*, 8 MIC indicates the dose of antibiotics with 8× MIC value of *P. aeruginosa,* PHAGE + antibiotic indicates simultaneous treatment and PHAGE 6 H + antibiotics indicates phage was added first then antibiotic was added with 6 h delay. * Under detection limit (<10^2^). (**^**) Statistical differences between the control and treated biofilms. (#) Statistical differences between the simultaneously and sequentially treated biofilms. Statistical differences were determined by two-way repeated-measures analysis of variance (ANOVA) with a Tukey’s multiple comparison test.

The killing capacity of gentamicin (MIC) and EPA1 was tested individually and in combination (simultaneous and sequential treatments) in a dual-species biofilm model comprising *P. aeruginosa* and *S. aureus* (Figure 6). Intact biofilms were grown for 48 h and treated for 24 h in total. In the control, it was possible to observe a predominance of *P. aeruginosa* (1.4 × 10^9^ CFU/mL), in comparison with *S. aureus* (2.3 × 10^5^ CFU/mL) (Figure 7). Although *S. aureus* was the first colonizer, CLSM images indicate that both species were randomly distributed throughout the biofilm 3D structures (Figure 6). 

**Figure 8:**
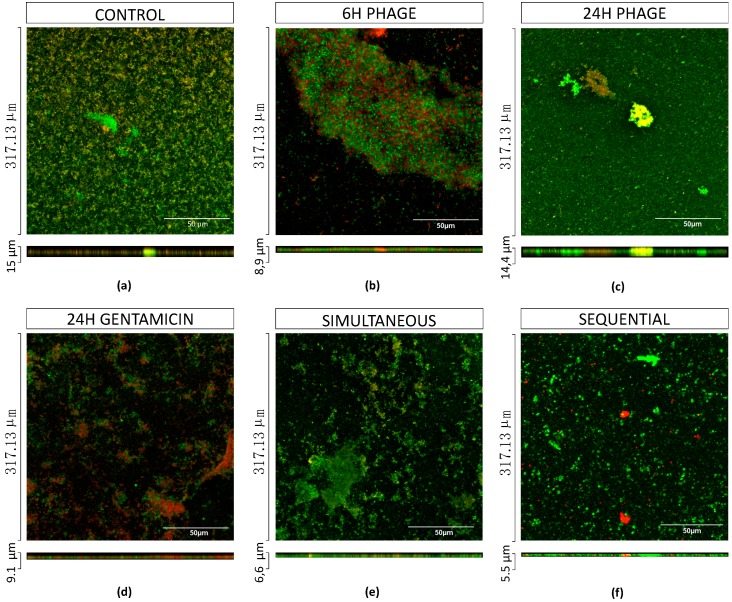
3D reconstructions of confocal stacks of images of dual-species of *P. aeruginosa* and *S. aureus* biofilms. (**a**) Control, (**b**) 6 h phage treatment, (**c**) 24 h phage treatment, (**d**) 24 h Gentamicin with MIC treatment, (**e**) 24 h simultaneous treatment, (**f**) 24 h sequential treatment. 48 h old intact biofilms were stained by using LM12_AMI-SH3 (with GFP) and EPA1_TFP (with mCherry) recombinant proteins. Scale bar represents 50 μm.

The individual treatments with gentamicin with MIC and 8× MIC resulted in a significant reduction of approximately 3.3 orders-of-magnitude and 4.6 orders-of-magnitude of *P. aeruginosa* cells, respectively. Phage treatment was less effective than gentamicin, resulting in a reduction of 0.7 orders-of-magnitude of *P. aeruginosa* cells (Figure 8b). None of the individual treatments showed a significant impact on the *S. aureus* population (Figure 7).

Regarding the simultaneous treatments, no synergy was observed for any combination. On *P. aeruginosa*, phage-gentamicin MIC resulted in 4.1 orders-of-magnitude reduction (additive effect), while the phage-gentamicin 8 × MIC only slightly increased the reduction (4.6 orders-of-magnitude), demonstrating an antagonistic effect (Figure 8d). Both treatments also had a positive effect on *S. aureus* biofilm cell control, significantly reducing CFUs by about 0.4 and 0.8 orders-of-magnitude when using phage-gentamicin MIC and phage-gentamicin 8xMIC treatments, respectively (Figure 7).

Similarly, in mono-species biofilms, a sequential treatment was also tested. Results indicate that a preliminary phage treatment (6 h) before gentamicin application was very effective in biofilm reduction. Phage-gentamicin MIC reduced about 6.3 orders-of-magnitude the *P. aeruginosa* population, while phage-gentamicin 8× MIC almost eradicated *P. aeruginosa* cells (approximately 7orders-of-magnitude reduction) (Figure 8f). Although the phage-gentamicin treatment did not significantly impact the *S. aureus* population, the phage-gentamicin 8× MIC sequential application was also the most efficient treatment in reducing *S. aureus* biofilm cells in about 2 orders-of-magnitude (Figure 7).

In order to infer the impact of combined treatments on the biofilm structure, CLSM observations were performed on dual-species biofilms before and after the treatments. In this case, as in mono-species biofilms, we also observed a reduction of biofilm thickness, from 15 μm to 8.9 μm after 6 h of phage infection, and a biofilm thickness increase to 14.4 μm after 24 h of phage infection. The significant biofilm thickness reductions were observed in simultaneous and sequential combined treatments, after which the remaining biofilms had thicknesses of 6.6 μm and 5.5 μm, respectively.

## 3. Discussion

EPA1 is a lytic *Pseudomonas* phage, isolated from a hospital sewage, that belongs to the *Myoviridae* family (Figure 1) This phage has no identifiable lysogeny-associated genes or genes coding for toxins (Table S2). Phages from *Pakpunavirus* genus are highly conserved genetically and are described as having a broad host range, being therefore described as good for therapy. Indeed, EPA1 presented similar features and therefore could be considered a good candidate for further phage therapy approaches [28].

Herein, this phage was studied for its potential synergistic activity in combinations (simultaneous and sequential) with different antibiotics (at MIC and 8× MIC) against mono and dual-species biofilms.

Initially, three antibiotics (gentamicin, ciprofloxacin and meropenem) belonging to different classes were tested on mono-species biofilms. The efficacy of antibiotics ranged from 0.8 to 5 orders-of-magnitude (Figure 2) on *P. aeruginosa* biofilms, and no significant reductions were observed for *S. aureus*, except when gentamicin was applied with 8× MIC which reduced the biofilm density by approximately 1.4 orders-of-magnitude (Figure S2). The reductions were of 3.4 and 0.5 orders-of-magnitude when EPA1 was applied individually for 6 h and 24 h on *P. aeruginosa* biofilms, respectively.

To augmenting the effect of antimicrobials, combined therapies were applied. Interestingly, a synergistic effect of phages and antibiotics in simultaneous combinations was only observed when antibiotics were applied at MIC (Figure 2). Surprisingly, the increased concentration of antibiotics (8xMIC) in simultaneous combinations have not resulted in a higher biofilm efficacy (Figure 2). The fact that the increase in antibiotic concentrations did not lead to an increase of the antibiofilm efficacy might be related to phage replication inhibition phenomena [18]. As phage particles are constituted mainly by proteins, protein synthesis inhibitors and DNA synthesis inhibitors might affect the formation of new phage particles and therefore have an antagonistic effect on phage replication [18,29]. Our results (Figure S3) corroborate that phage titers obtained after phage infection in combination with antibiotic treatments were significantly lower than the titer of phage in the control (phage infection without antibiotic treatment).

Phage-antibiotic sequential approaches were already reported as a promising antibiofilm strategy [30]. In this study, an impressive biofilm biomass reduction was obtained when antibiotics were added to biofilms after 6 h of phage treatment. This was the time point where phages caused the maximum biofilm reduction. Similar observations were already reported elsewhere [27]. When phages interact with *P. aeruginosa* biofilms an initial population reduction is observed up to 6 h of phage contact and after that period the biofilm regrows. Biofilm regrowth was attributed to the emergence of phage-resistant variants that were equally well adapted to the biofilm phenotype [27].

On sequential treatments, we observed a synergistic effect with all combinations, when antibiotics were applied at their MICs. However, the antibiotic that showed the best antibiofilm efficacy was gentamicin. The combined treatment with this antibiotic was enough to eliminate all detectable biofilm cells in a concentration-dependent manner. The same phenomenon was not observed when other four protein synthesis inhibitors were used (Figure 4).

The antimicrobial synergy between phages has been in part explained by a more efficient penetration of both antibiotics and phages into the biofilm. In fact, it has been described that phages can degrade the biofilm matrix using depolymerases [31,32], enhancing their penetration into the deep layers of the biofilm. In the case of EPA1, no depolymerase was identified in its genome, therefore we have no evidence that this phenomenon might have been responsible for a synergistic action of the combined therapy. We have previously reported that phages can access the bottom layers of the biofilm migrating through the biofilm void spaces [33]. This might be the case in our study, since both mono and dual-species biofilms appeared to be heterogeneous structures with plenty of void spaces (Figure 3 and Figure 6). This phenomenon leads phages to replicate in the deeper-layer of biofilm, reaching high titers and interrupting the biofilm matrix. The addition of antibiotics following this interruption results in an enhanced bacterial reduction due to the deeper penetration of these agents. When phages are applied prior to the antibiotic, they also avoid the antagonistic effect of antibiotics on phage replication, as was previously described [18]. After determining the best-case scenario to treat *P. aeruginosa* biofilms, it was our intention to study the impact of this treatment on dual-species biofilms. The relevance of studying polymicrobial communities has gained more interest on the last decade, mainly due to the fact that the vast majority of biofilms are formed by more than one species of bacteria and in some case also fungi, protozoa and algae [34]. This reason triggered the design of this study, and to our knowledge, this is the first time that this strategy is reported in dual-species biofilms.

The establishment of dual-species biofilm models was difficult, due to the inhibitory effect of *P. aeruginosa* on *S. aureus* cells, which is widely reported [9,10,35,36]. It is described that polymicrobial biofilms of chronic wounds are first colonized by small numbers of resident Gram-positive aerobic cocci, including *S. aureus*, after which there is a shift on wound microbiome, and Gram-negative bacilli are predominant on this environment [37]. As previously performed by DeLeon et al. [26], in our experiments, dual-species biofilms were established in two-steps. Colonization by *S. aureus* and a further addition of *P. aeruginosa* led to the establishment of stable biofilms.

In general, dual-species biofilms were more tolerant to all treatments than mono-species *P. aeruginosa* biofilms. Nevertheless, the sequential treatment at 8× MIC almost eradicated *P. aeruginosa* biofilm cells, but it did not increase the antimicrobial effect on *S. aureus*.

The presence of external selective pressures, like antibiotics or phages, can stimulate a cell response that can lead to increased tolerance to antibiotics, namely as a result of EPS production by *P. aeruginosa*. Alginate, Pel, and Psl, which are part of the biofilm extracellular matrix have a structural and protective function [38,39]. More specifically, Psl is described as creating a protective barrier against aminoglycosides [40]. Also, quorum-sensing molecules can justify the weaker effect of these approaches on dual-species biofilms [41].

The data collected herein showed that the concentration of gentamicin needs to be increased (8× MIC) to eliminate *P. aeruginosa* cells successfully in dual-species biofilms in comparison with mono-species biofilms. This can result in an overdose of antibiotics that can lead to toxicity in treatments [42].

Overall, from this study, two main conclusions emerge. First, the combined treatment with a sequential application of phages and then antibiotic is the most promising approach to combat infectious biofilms when compared with their individual and simultaneous treatments. Second, the majority of the studies of antibiofilm approaches are conducted in mono-species biofilms, and as demonstrated herein, the treatment outcomes are completely different when a second species is added. So, to achieve success, a phage cocktail comprising different *P. aeruginosa* and *S. aureus* phages targeting different bacterial receptors should be tested prior to antibiotic addition.

## 4. Material and Methods

### 4.1. Bacterial Strains and Culture Conditions

The biofilm-forming strains *P. aeruginosa* PAO1 and *S. aureus* (ATCC^®^25923™) were obtained from LPhage Laboratory in the Centre of Biology (CEB) strain collection (Braga, Portugal). Additional one *P. aeruginosa* clinical isolate and one Spanish Type Culture Collection (CECT) strain were obtained from Lphage Laboratory in CEB strain collection and Sifa Hospital Strain collection (Izmir, Turkey). In total, this accounts for a total of 20 strains used (Table S1) for isolating and characterizing the phage. The antibiogram profile of the clinical strains was previously established by the provider institutes. All strains were grown in Tryptic Soy Broth (TSB, VWR Chemicals, Randor, PA, USA), Tryptic Soy Agar (TSA; VWR Chemicals) or in TSA soft overlays (TSB with 0.6% agar) at 37 °C. In addition, mannitol salt agar (MSA; VWR Chemicals) was used to enumerate *S. aureus* cells in dual-species biofilms, while *P. aeruginosa* cells were counted in non-selective media (TSA).

### 4.2. Phage Isolation and Production

Phage was isolated from effluent samples of raw sewage from Sifa Hospitals in Izmir, Turkey. The phage enrichment method was applied to isolate the phage [43]. Briefly, 100 mL of the effluent were mixed with 100 mL of double-strength TSB and with 10 µL of each of the exponentially grown *P. aeruginosa* strains. The obtained suspensions were incubated at 37 °C and 120 rpm (BIOSAN ES-20/60, Riga, Latvia) overnight. Suspensions were further centrifuged (15 min, 9000× *g*, 4 °C), and the supernatants were filtered through a 0.22 µm polyethersulfone (PES) membrane (ThermoFisher Scientific, Massachusetts, USA). The presence of phages was confirmed by performing spot assays on bacterial lawns. The prepared plates were further incubated overnight at 37 °C, and the presence of inhibition halos observed. When phage plaques appeared, successive rounds of single plaque purification were carried out until purified plaques were observed, reflected by a single plaque morphology.

The purified phage was produced by using the double agar layer method, as described before [43]. Briefly, 100 µL of a phage suspension at 10^8^ PFU/mL were spread on *P. aeruginosa* PAO1 lawns for overnight incubation at 37 °C. If full lysis was observed, plates were further incubated at 4 °C for 6 h at 120 rpm (BIOSAN PSU-10i), with 2 mL of SM Buffer (100 mM NaCl, 8 mM MgSO4, 50 mM Tris/HCl, pH 7.5) to resuspend the phage particles. The liquid phase was collected and centrifuged (15 min, 9000× *g*, 4 °C), and the supernatants were filtered through a 0.22 µm PES membrane. Purified phages were stored at 4 °C for further use.

### 4.3. Electron Microscopy

Phage suspension was sedimented by centrifugation (25,000× *g*, 60 min, 4 °C) using a ScanSpeed 1730R centrifuge (Labogene, Lillerød, Denmark). The pellet was further washed in tap water by repeating the centrifugation step. Subsequently, phage suspension was deposited on copper grids with a carbon-coated Formvar carbon film on a 200 square mesh nickel grid, stained with 2% uranyl acetate (pH 4.0) and examined using a Jeol JEM 1400 transmission electron microscope (TEM) (Tokyo, Japan).

### 4.4. Phage Host Range and Efficiency of Plating Determination

Phage host range was determined on the strains listed in Table 3, using the spot test method [43]. Briefly, 100 μL of each host-growing culture were added to 3 mL of TSB-soft agar and poured onto TSB agar plates. The bacterial lawns were spotted with 10 μL of serial 10-fold dilutions of the phage suspension and incubated at 37 °C for overnight and results were analyzed. The EOP was calculated by dividing the titer of the phage (PFU/mL) obtained in each isolate by the titer determined in the propagating host. EOP was recorded as high (>10%), moderate (0.01–9%) or low (<0.01%) [43].

### 4.5. Genome Sequencing and in Silico Analysis

*P. aeruginosa* EPA1 genomic DNA was extracted according to the standard methods with phenol-chloroform-isoamyl alcohol, as described elsewhere [44]. The genome was sequenced using the genome sequencer FLX Instrument (Roshe Life Science), *de novo* assembled using Geneious R9 and manually inspected. The genome was annotated using MyRAST algorithm [45]. The CDSs putative functions were assigned using BLASTP [46] with tRNAs being predicted with tRNAscan-SE [47] and ARAGORN [48]. HHPRED [49] was used to detect protein homology and structure prediction. N-terminal signal peptides with SignalP 3.0. [50] Transcriptional factors were determined by MEME [51] and ARNold [52] for the promoter and rho-independent terminators, respectively. For comparative studies, genomic comparisons were made using BLASTN and OrthoVenn [53], for DNA and protein sequence similarities.

### 4.6. Minimal Inhibitory Concentration Determination

Seven different antibiotics were selected to use in the study: gentamicin, kanamycin, tetracycline, chloramphenicol, erythromycin, ciprofloxacin and meropenem. MIC values were determined by the microdilution method for *P. aeruginosa* PAO1 according to the described method [54] (Table 2).

### 4.7. Establishing Mono and Dual-Species Biofilms

Mono and dual-species biofilm formation was performed in 24 polystyrene well plates (Orange Scientific, Braine-l’Alleud, Belgium). For initiating the biofilms, one bacterial colony (*P. aeruginosa* or *S. aureus)* was incubated in TSB overnight in an orbital shaker (120 rpm, BIOSAN ES-20/60) at 37 °C.

For establishing mono-species biofilms, 10 µL of the starter culture were transferred into 24-well plates containing 990 µL of fresh TSB media. The plates were incubated for 24 h in an orbital shaker incubator (120 rpm, BIOSAN ES-20/60) at 37 °C. After 24 h, half of the growth media (500 µL TSB, 1:1, *v:v*) was replaced with fresh TSB then, incubated for more 24 h.

For dual-species biofilms, the procedure was similar with some differences. *S. aureus* cells were inoculated prior to *P. aeruginosa* addition. Thus, biofilms were initiated with 10 µL of the overnight culture of *S. aureus* (~10^8^ CFU/mL) in 990 µL TSB and incubated for 24 h in an orbital shaker (120 rpm) at 37 °C. After that, half of the growth media (500 µL TSB, 1:1, *v:v*) was replaced with TSB including 10 µL of the starter culture of *P. aeruginosa* (~10^8^ CFU/mL, 1:49, *v/v*) and incubated for additional 24 h.

In mono and dual-species biofilms, the liquid part was aspirated, and the wells were washed twice with saline solution (0.9% NaCl (*w/v*)) to remove planktonic bacteria. Biofilms were further scraped in saline solution (1 mL) using a micropipette tip, and the number of viable cells was determined using the microdrop method [43]. Three independent experiments were performed in duplicate.

### 4.8. Biofilm Challenge

Forty-eight hours old mono and dual-species biofilms were treated with the antimicrobials; individually, in simultaneous or sequential combinations for 24 h post-treatment. Briefly, biofilms were washed twice with the saline solution and antibiotics (MIC or 8× MIC) and phage (MOI 1) were applied in TSB, individually. Also, the efficacy of two combinations was tested. In simultaneous combination, one of the selected antibiotics with MIC or 8× MIC combined with phage at MOI 1 in TSB solution was added into biofilm-bearing wells for 24 h post-treatment. In sequential combination, phage at MOI 1 was added into biofilm-bearing wells for 6 h, and then one of the antibiotics (final concentration of MIC or 8× MIC) was added into the well plates for additional 18 h. The reverse sequential combination, gentamicin at MIC and 8× MIC were added into biofilm wells for 6 h, and then phage EPA1 with MOI 1 was added into the well plates for additional 18 h. The number of viable cells was enumerated using the microdrop method [43].

The potential interaction of treatments with biofilms is described as synergistic when the biofilm reduction in combinations is greater than the sum of individual treatments of antimicrobials, as described by Chaudhry et al. [18]. An interaction is described as an additive when the biofilm reduction in combinations is similar/equal to the sum of individual treatments of antimicrobials. An interaction is described as antagonistic when the biofilm reduction in combinations is lower than the sum of individual treatments of antimicrobials. The efficacy of treatment can be defined according to the result of the following equations:

log (AP) − ((log (A) + log (P))

=0, additive interaction

>0, Synergistic interaction

<0, Antagonistic interaction

In which P is the reduction in the number of viable biofilm cell in individual phage treatment; A the reduction in the number of viable biofilm cell in individual antibiotic treatment; AP the reduction in the number of viable biofilm cell in combined treatment.

### 4.9. Development of Probes for Biofilm Imaging 

To assess the structure of biofilm models, bacteria-specific fluorescent probes were constructed using phage proteins. Given the expertise of our laboratory on phage-based protein construction, this method was selected instead of the use of commercial probes in the biofilm imaging process.

The red fluorescent mCherry gene derived from the DsRed of *Discosoma* sea anemones was inserted into the plasmid pET28a (+) (Novagen, Merck, Darmstady, Germany), between the *Sac*I and *Xho*I restriction sites conserving the plasmid N-terminal hexahistidine (His)-tag sequence and originating the pET_mCherry plasmid. Primers were designed to obtain fragments of the EPA1_gp81 tail fiber C-terminus (further referred to as the EPA1_TFP with mCherry). The fragments were amplified with Phusion DNA Polymerase (ThermoFisher Scientific) with the EPA1 genome as DNA template and digested with the restriction enzymes *Sac*I and *Xho*I. The digested fragments were inserted into pET28a(+) and ligated with the T4 ligase (ThermoFisher Scientific) to obtain the construction (pET_ EPA1_TFP), further used to transform *E. coli* TOP10 competent cells (Invitrogen, California, USA). Colonies were screened through colony PCR and positives used for plasmid extraction and further confirmation through Sanger sequencing. A correct pET_EPA1_TFP plasmid was used to transform competent *E. coli* BL21. Besides, the *S. aureus* phage vB_SauM-LM12 [43] endolysin truncated at its N-terminus and fused with GFP (LM12_AMI-SH3 with GFP) was constructed by our group [55].

Expression of the different peptides was performed as described before [56]. Briefly, the cells harboring recombinant plasmids were grown at 37 °C in Lysogeny Broth (LB) supplemented with 50 μg/mL of kanamycin until reaching an optical density at 620 nm (OD_620nm_) of 0.6. Recombinant protein expression induced with isopropyl-β-D-thiogalactopyranoside (IPTG; Thermo Fisher Scientific) at 1 mM final concentration was carried overnight at 16 °C, 150 rpm. Cells were collected by centrifugation (9000× *g*, 15 min, 4 °C) and further resuspended in lysis buffer (20 mM NaH_2_PO_4_, 500 mM sodium chloride, 10 mM imidazole, pH 7.4). Cell disruption was made by thaw-freezing (3 cycles, from −80 °C to room temperature) followed by a 5 min sonication (Cole-Parmer Ultrasonic Processor) for 10 cycles (30 s ON, 30 s OFF), 40% amplitude. Soluble cell-free extracts were separated by centrifugation, filtered, and loaded on a 1 mL HisPur™Ni-NTA Resin (Thermo Fisher Scientific) stacked into a Polypropylene column (Qiagen). After two washing steps with protein-dependent imidazole concentrations (lysis buffer supplemented with 20 mM imidazole in the first wash, and 40 mM imidazole in the second wash), the protein was eluted with 300 mM imidazole. Protein fractions were observed through SDS-PAGE. The purified proteins were quantified using the Pierce^TM^ BCA Protein Assay Kit (Thermo Ficher).

### 4.10. CLSM Analysis

CLSM was performed as described before [56] with some modifications. Briefly, the 13 mm in diameter Thermanox^®^ Plastic Coverslip (Rochester, New York, USA) were placed in 24-well plates, and mono and dual-biofilms were formed as mentioned before. Coverslips were further washed twice with saline solution, and treatments were applied. After the treatment, the suspension was aspirated, and the wells were washed twice with 0.9% saline solution. The fluorescence probes, EPA1_TFP with mCherry (laser excitation line 635nm and emissions filters BA 655–755, red channel) and LM12_AMI-SH3 with GFP (laser excitation line 488 nm and emissions filters BA 505–605, green channel) were used for detection of cells in biofilms. The coupons were stained with 15 µL of probes in a final concentration of 20 mM for 15 min. After, the images were acquired in a Confocal Scanning Laser Microscope (Olympus B × 61, Model FluoView 1000) with the program FV10-Ver4.1.1.5 (Olympus). For each condition, three independent biofilms were used.

### 4.11. Statistical Analysis

The results of assays were compared using two-way analysis of variance (ANOVA) by applying the Tukey’s multiple comparisons test using Prism 6 (GraphPad, La Jolla, CA, USA). Means and standard deviations (SD) were calculated with the software. Differences among conditions were considered statistically significant when *p* < 0.001.

### 4.12. Nucleotide Sequence Accession Number

The genome sequence of *Pseudomonas* phage vB_PaM_EPA1 was deposited in the GenBank database under the accession number MN013356.

## Figures and Tables

**Table 1:** General features of EPA1 genome.

Feature	vB_PaM_EPA1
Genome size	91,394 bp
G+C content	49,2%
Number of predicted CDSs	175
Number of proteins with assigned functions	35

**Table 2:** List of the antibiotics, MIC values of *P. aeruginosa* and *S. aureus* planktonic cells and their mechanism of action.

Name of Antibiotics	*P. aeruginosa* MIC Values	*S. aureus* MIC Values	Mechanism of Action	
Gentamicin	4 µg/mL	16 µg/mL	Protein Synthesis Inhibitors	30S ribosomal subunit
Kanamycin	10 µg/mL	*	Protein Synthesis Inhibitors	
Tetracycline	8 µg/mL	*	Protein Synthesis Inhibitors	
Chloramphenicol	32 µg/mL	*	Protein Synthesis Inhibitors	50S ribosomal subunit
Erythromycin	128 µg/mL	*	Protein Synthesis Inhibitors	
Ciprofloxacin	<1 µg/mL	<1 µg/mL	DNA Synthesis Inhibitor	
Meropenem	2 µg/mL	2 µg/mL	Cell wall Synthesis Inhibitor	

* These antibiotics were not tested on *S. aureus* biofilm models.

**Table 3:** General overview of the efficacy of combined treatments in 48 h *P. aeruginosa* mono-species biofilm. Synergistic—the biofilm reduction using phage-antibiotic combinations is greater than the sum of their individual treatments. Additive—the biofilm reduction using phage-antibiotic combination is similar to the sum of their individual treatments. Antagonistic—the biofilm reduction using phage-antibiotic combinations is lower than the sum of their individual treatments.

Treatments	Gentamicin	Ciprofloxacin	Meropenem
Simultaneously MIC	Synergistic	Synergistic	Synergistic
Simultaneously 8 MIC	Additive	Antagonistic	Antagonistic
Sequentially MIC	Synergistic	Synergistic	Synergistic
Sequentially 8 MIC	Synergistic	Synergistic	Antagonistic

## Supplementary Materials

The following are available online at https://www.mdpi.com/2079-6382/8/3/103/s1, Figure S1. One-step growth curve of phage vB_PaM_EPA1 in *P. aeruginosa* Sifa_Pa_1.5 at 37 °C. Shown are the PFU per infected cell; Figure S2. Treatment of *S. aureus* 48 h biofilms population with different antimicrobial agents (gentamicin, ciprofloxacin and meropenem) for 24 h; Figure S3. The effect of the antibiotics (gentamicin, ciprofloxacin and meropenem) on phage replication for 24 h post-treatment; Figure S4. Treatment of *P. aeruginosa* PAO1 48 h biofilms population with reverse sequential combinations. Table S1. Bacterial strains feature and susceptibility to phage EPA1; Table S2. The genome annotation of phage vB_PaM_EPA1.
