# Supplementary material for: Synergistic Action of Phage and Antibiotics: Parameters to Enhance the Killing Efficacy Against Mono and Dual-Species Biofilms
Source: Antibiotics (Basel). 2019 Jul 25;8(3):103. doi: 10.3390/antibiotics8030103 (PMC6783858; doi:10.3390/antibiotics8030103)
Supplement: Supplementary file 1 [file antibiotics-08-00103-s001.zip › Supplementary File/20190613 -Supplementary File - Synergistic action of phage and antibiotics.docx]

Article

Synergistic action of phage and antibiotics: Parameters to enhance the killing efficacy against mono and dual-species biofilms

Ergun Akturk^1^, Hugo Oliveira^2^, Silvio B. Santos^3^, Luis DR Melo^4^* and Joana Azeredo ^5^*

^1^ LIBRO-Laboratório de Investigação em Biofilmes Rosário Oliveira, Centre of Biological Engineering, University of Minho, Campus de Gualtar, 4700-057, Braga, Portugal. ergun.akturk@ceb.uminho.pt.

^2^ LIBRO-Laboratório de Investigação em Biofilmes Rosário Oliveira, Centre of Biological Engineering, University of Minho, Campus de Gualtar, 4700-057, Braga, Portugal. hugo.oliveira@deb.uminho.pt.

^3^ LIBRO-Laboratório de Investigação em Biofilmes Rosário Oliveira, Centre of Biological Engineering, University of Minho, Campus de Gualtar, 4700-057, Braga, Portugal. silviosantos@ceb.uminho.pt.

^4^ LIBRO-Laboratório de Investigação em Biofilmes Rosário Oliveira, Centre of Biological Engineering, University of Minho, Campus de Gualtar, 4700-057, Braga, Portugal. lmelo@deb.uminho.pt.

^5^ LIBRO-Laboratório de Investigação em Biofilmes Rosário Oliveira, Centre of Biological Engineering, University of Minho, Campus de Gualtar, 4700-057, Braga, Portugal. jazeredo@deb.uminho.pt.

***** Correspondence: lmelo@deb.uminho.pt and jazeredo@deb.uminho.pt.

**Table S1.** Bacterial strains features and susceptibility to phage EPA1.

| No | Name of Strain | Source | Country | Infectivity | Antibiotic Resistance* | EOP** |
| --- | --- | --- | --- | --- | --- | --- |
| 1 | Sifa_Pa_1.5^a^ | Abscess | Turkey | SXT, FOX, CXM, AMP, CXA, AMC | + | High |
| 2 | Sifa_Pa_1.8 | Sputum | Turkey | CAZ, IMP, CIP, TZP, PRL | + | Moderate |
| 3 | Sifa_Pa_1.9 | Sputum | Turkey | SXT, FOX, CXM, AMP, CXA | - |  |
| 4 | Sifa_Pa_1.12 ^a^ | Blood | Turkey | SXT, TZP | - |  |
| 5 | Sifa_Pa_1.13 | Sputum | Turkey | CAZ, CN | + | High |
| 6 | Sifa_Pa_1.17 ^a^ | Lavage | Turkey | CT, SXT | + | Low |
| 7 | Sifa_Pa_1.3 | Wound | Turkey | SXT, FOX, CXM, AMP, CXA, AMC | - |  |
| 8 | Sifa_Pa_1.15 ^a^ | Adenoid | Turkey | SXT, FOX, CXM, AMP, CXA, AMC | + | High |
| 9 | Sifa_Pa_1.9 | Wound | Turkey | IPM, CT, SXT | - |  |
| 10 | Sifa_Pa_1.4 | Wound | Turkey | FOX, CXM, SAM, AMP, CXA | - |  |
| 11 | Sifa_Pa_149 | Catheter Tip | Turkey | CN, AN, SXT, SAM, TGC, NET, TE | + | Moderate |
| 12 | Sifa_Pa_148 | Sputum | Turkey | SXT, TZP | + | High |
| 13 | Sifa_CuPa_001 | Blood | Turkey | FOX, AMP, CXA | + | High |
| 14 | Sifa_CuPa_002 | Blood | Turkey | SXT, CXM, CXA | + | High |
| 15 | Sifa_CuPa_003 ^a^ | Blood | Turkey | FOX, AMP | + | Moderate |
| 16 | Sifa_CuPa_004 | Blood | Turkey | SXT, CXM, AMP, CXA | + | High |
| 17 | MiPa_025 ^a^ | Unknown | Portugal | IMP, CIP, CAZ, GM, TZP | + | High |
| 18 | *P. aeruginosa* PAO1 |  |  |  | + | High |
| 19 | *P. aeruginosa* ATCC 10145 |  |  |  | + | High |
| 20 | *P. aeruginosa* CECT 111 |  |  |  | - |  |

* Name of Antibiotics: Ceftazidime (CAZ), Imipenem (IPM), Ciprofloxacin (CIP), Gentamicin (GEN), Amikacin (AN), Colitis (CT), Co-trimoxazole (SXT), Cefoxitin (FOX), Cefuroxime (CXM), Ampicillin-sulbactam (SAM), Ampicillin (AMP), Cefuroxime axetil (CXM), Piperacillin-Tazobactam (TZP), Piperacillin (PIP), Amoxicillin/clavulanate (AMC), Levofloxacin (LVX), Tigecycline (TGC), Netilmicin (NET), Tetracycline (TET), Cefoperazone sulbactam (SCF).

****** The EOP was determined as high, moderate, low and lysis from without (LFW). +: EPA1 has lytic activity against target host. – : EPA1 has not lytic activity against target host. EOP: efficiency of plating

^a^: Used for phage isolation

**Table S2.** Features of phage EPA1 predicted CDSs. For each CDS the transcription starts and stop position, and the coding strand and direction are given. At protein level the corresponding gene product size molecular weight and pH as well as the homolog predicted function homology, accession number and values are shown.

| **ORF** | **Direction**  **(←, →)** | **Start (bp)** | **Stop (bp)** | **Size (aa)** | **MW (kDa)** | **PH** | **Best Species Hit** | **Putative Function** | **Accession number** | **E-value (% identify)** |  |
| --- | --- | --- | --- | --- | --- | --- | --- | --- | --- | --- | --- |
| EPA1_01 | ← | 24 | 161 | 138 | 5.02 kDa | pH 6.49 | hypothetical protein PaP1_gp156 [Pseudomonas phage PaP1] | hypothetical protein CDS | [YP_007236567.1](http://www.ncbi.nlm.nih.gov/protein/431809674?report=genbank&log$=prottop&blast_rank=1&RID=5W5MS414014) | 6.00E-24 | |
| EPA1_02 | → | 346 | 939 | 594 | 22.80 kDa | pH 8.21 | hypothetical protein [Pseudomonas phage vB_PaeM_C2-10_Ab02] | hypothetical protein CDS | [CEF88932.1](http://www.ncbi.nlm.nih.gov/protein/713662090?report=genbank&log$=prottop&blast_rank=1&RID=5W5MS414014) | 1.00E-144 | |
| EPA1_0002A | → | 1,026 | 1,169 | 144 | 5.29 kDa | pH 12.52 | hypothetical protein PJG4_002 [Pseudomonas phage JG004] | hypothetical protein CDS | [YP_007002526.1](http://www.ncbi.nlm.nih.gov/protein/418488229?report=genbank&log$=prottop&blast_rank=1&RID=5W5MS414014) | 2.00E-14 | |
| EPA1_0003 | → | 1,447 | 1,596 | 150 | 5.01 kDa | pH 4.67 | hypothetical protein PJG4_003 [Pseudomonas phage JG004] | hypothetical protein CDS | [YP_007002525.1](http://www.ncbi.nlm.nih.gov/protein/418488230?report=genbank&log$=prottop&blast_rank=1&RID=5W5MS414014) | 4.00E-25 | |
| EPA1_0004 | ← | 1,659 | 1,925 | 267 | 10.64 kDa | pH 4.78 | hypothetical protein PJG4_004 [Pseudomonas phage JG004] | hypothetical protein CDS | [YP_007002524.1](http://www.ncbi.nlm.nih.gov/protein/418488231?report=genbank&log$=prottop&blast_rank=1&RID=5W5MS414014) | 9.00E-59 | |
| EPA1_0005 | ← | 1,925 | 2,329 | 405 | 15.30 kDa | pH 4.95 | hypothetical protein PAK_P200127c [Pseudomonas phage PAK_P2] | hypothetical protein CDS | [YP_008857168.1](http://www.ncbi.nlm.nih.gov/protein/563397403?report=genbank&log$=prottop&blast_rank=1&RID=5W5MS414014) | 1.00E-94 | |
| EPA1_0006 | ← | 2,319 | 2,735 | 417 | 16.03 kDa | pH 4.52 | hypothetical protein [Pseudomonas phage vB_PaeM_C2-10_Ab08] | hypothetical protein CDS | [CEF89320.1](http://www.ncbi.nlm.nih.gov/protein/713662512?report=genbank&log$=prottop&blast_rank=1&RID=5W5MS414014) | 2.00E-97 | |
| EPA1_0007 | ← | 2,790 | 3,464 | 675 | 26.36 kDa | pH 8.63 | hypothetical protein PAK_P200129c [Pseudomonas phage PAK_P2] | hypothetical protein CDS | [YP_008857170.1](http://www.ncbi.nlm.nih.gov/protein/563397405?report=genbank&log$=prottop&blast_rank=1&RID=5W5MS414014) | 6.00E-158 | |
| EPA1_0008 | ← | 3,467 | 3,745 | 279 | 10.58 kDa | pH 10.23 | hypothetical protein PaP1_gp006 [Pseudomonas phage PaP1] | hypothetical protein CDS | [YP_007236417.1](http://www.ncbi.nlm.nih.gov/protein/431809524?report=genbank&log$=prottop&blast_rank=1&RID=5W5MS414014) | 2.00E-53 | |
| EPA1_0009 | ← | 3,733 | 4,044 | 312 | 11.95 kDa | pH 9.97 | hypothetical protein PAK_P400130c [Pseudomonas phage PAK_P4] | hypothetical protein CDS | [YP_008859342.1](http://www.ncbi.nlm.nih.gov/protein/563399501?report=genbank&log$=prottop&blast_rank=1&RID=5W5MS414014) | 2.00E-66 | |
| EPA1_0010 | ← | 4,044 | 4,460 | 417 | 15.62 kDa | pH 4.00 | hypothetical protein K8_011 [Pseudomonas phage K8] | hypothetical protein CDS | [ALF51333.1](http://www.ncbi.nlm.nih.gov/protein/930348886?report=genbank&log$=prottop&blast_rank=1&RID=5W5MS414014) | 4.00E-53 | |
| EPA1_0011 | ← | 4,457 | 4,741 | 285 | 11.47 kDa | pH 4.44 | hypothetical protein PAK_P400132c [Pseudomonas phage PAK_P4] | hypothetical protein CDS | [YP_008859344.1](http://www.ncbi.nlm.nih.gov/protein/563399503?report=genbank&log$=prottop&blast_rank=1&RID=5W5MS414014) | 3.00E-44 | |
| EPA1_0012 | ← | 4,796 | 5,011 | 216 | 8.36 kDa | pH 4.11 | hypothetical protein PAK_P100139c [Pseudomonas phage PAK_P1] | hypothetical protein CDS | [YP_004327153.1](http://www.ncbi.nlm.nih.gov/protein/326804349?report=genbank&log$=prottop&blast_rank=1&RID=5W5MS414014) | 6.00E-44 | |
| EPA1_0013 | ← | 5,008 | 5,349 | 342 | 12.58 kDa | pH 4.50 | hypothetical protein PAK_P100140c [Pseudomonas phage PAK_P1] | hypothetical protein CDS | [YP_008869205.1](http://www.ncbi.nlm.nih.gov/protein/563448587?report=genbank&log$=prottop&blast_rank=1&RID=5W5MS414014) | 8.00E-76 | |
| EPA1_0014 | ← | 5,351 | 5,854 | 504 | 19.39 kDa | pH 5.34 | hypothetical protein [Pseudomonas phage vB_PaeM_C2-10_Ab02] | hypothetical protein CDS | [CEF88945.1](http://www.ncbi.nlm.nih.gov/protein/713662103?report=genbank&log$=prottop&blast_rank=1&RID=5W5MS414014) | 4.00E-118 | |
| EPA1_0015 | ← | 5,841 | 6,218 | 378 | 14.92 kDa | pH 4.65 | hypothetical protein PAK_P200137c [Pseudomonas phage PAK_P2] | hypothetical protein CDS | [YP_008857178.1](http://www.ncbi.nlm.nih.gov/protein/563397413?report=genbank&log$=prottop&blast_rank=1&RID=5W5MS414014) | 5.00E-87 | |
| EPA1_0016 | ← | 6,238 | 6,819 | 582 | 21.51 kDa | pH 8.22 | hypothetical protein [Pseudomonas phage vB_PaeM_C2-10_Ab08] | DNA processing protein DprA CDS | [CEF89330.1](http://www.ncbi.nlm.nih.gov/protein/713662522?report=genbank&log$=prottop&blast_rank=1&RID=5W5MS414014) | 1.00E-139 | |
| EPA1_0017 | ← | 6,816 | 7,127 | 312 | 12.06 kDa | pH 4.45 | hypothetical protein [Pseudomonas phage vB_PaeM_C2-10_Ab08] | hypothetical protein CDS | [CEF89331.1](http://www.ncbi.nlm.nih.gov/protein/713662523?report=genbank&log$=prottop&blast_rank=1&RID=4VVW0JVV015) | 2.00E-69 | |
| EPA1_0018 | ← | 7,129 | 7,278 | 150 | 5.68 kDa | pH 10.37 | hypothetical protein PJG4_015 [Pseudomonas phage JG004] | hypothetical protein CDS | [YP_007002513.1](http://www.ncbi.nlm.nih.gov/protein/418488242?report=genbank&log$=prottop&blast_rank=1&RID=4VVW0JVV015) | 2.00E-25 | |
| EPA1_0019 | ← | 7,290 | 7,769 | 480 | 19.39 kDa | pH 5.34 | hypothetical protein [Pseudomonas phage vB_PaeM_C2-10_Ab02] | hypothetical protein CDS | [CEF88950.1](http://www.ncbi.nlm.nih.gov/protein/713662108?report=genbank&log$=prottop&blast_rank=1&RID=4VVW0JVV015) | 9.00E-113 | |
| EPA1_0020 | ← | 7,766 | 7,963 | 198 | 14.92 kDa | pH 4.65 | hypothetical protein PJG4_017 [Pseudomonas phage JG004] | hypothetical protein CDS | [YP_007002511.1](http://www.ncbi.nlm.nih.gov/protein/418488244?report=genbank&log$=prottop&blast_rank=1&RID=4VVW0JVV015) | 1.00E-40 | |
| EPA1_0021 | ← | 7,975 | 9,663 | 1,689 | 21.51 kDa | pH 8.22 | putative Nicotinamide phosphoribosyltransferase [Pseudomonas phage vB_PaeM_C2-10_Ab02] | Nicotinamide phosphoribosyltransferase CDS | [CEF88952.1](http://www.ncbi.nlm.nih.gov/protein/713662110?report=genbank&log$=prottop&blast_rank=1&RID=4VVW0JVV015) | 0 | |
| EPA1_0022 | ← | 9,720 | 9,938 | 219 | 12.06 kDa | pH 4.45 | hypothetical protein [Delftia] | hypothetical protein CDS | [WP_034365756.1](http://www.ncbi.nlm.nih.gov/protein/736339405?report=genbank&log$=prottop&blast_rank=1&RID=4VVW0JVV015) | 1.00E-43 | |
| EPA1_0023 | ← | 9,935 | 10,801 | 867 | 5.68 kDa | pH 10.37 | putative ribose-phosphate pyrophosphokinase [Pseudomonas phage PAK_P4] | Ribose-phosphate pyrophosphokinase CDS | [YP_008859356.1](http://www.ncbi.nlm.nih.gov/protein/563399515?report=genbank&log$=prottop&blast_rank=1&RID=4VVW0JVV015) | 0 | |
| EPA1_0024 | ← | 10,811 | 11,227 | 417 | 17.71 kDa | pH 4.56 | hypothetical protein PAK_P400145c [Pseudomonas phage PAK_P4] | hypothetical protein CDS | [YP_008859357.1](http://www.ncbi.nlm.nih.gov/protein/563399516?report=genbank&log$=prottop&blast_rank=1&RID=4VVW0JVV015) | 3.00E-97 | |
| EPA1_0025 | ← | 11,238 | 12,155 | 918 | 7.76 kDa | pH 9.83 | putative RNA ligase/tail attachment protein [Pseudomonas phage vB_PaeM_C2-10_Ab1] | RNA ligase/tail attachment protein | [YP_007236842.1](http://www.ncbi.nlm.nih.gov/protein/431809954?report=genbank&log$=prottop&blast_rank=2&RID=4VVW0JVV015) | 0 | |
| EPA1_0026 | ← | 12,167 | 12,574 | 408 | 62.79 kDa | pH 4.71 | hypothetical protein BN405_2-10_Ab1_orf_22 [Pseudomonas phage vB_PaeM_C2-10_Ab1] | hypothetical protein CDS | [YP_007236843.1](http://www.ncbi.nlm.nih.gov/protein/431809955?report=genbank&log$=prottop&blast_rank=1&RID=4VVW0JVV015) | 3.00E-89 | |
| EPA1_0027 | ← | 12,571 | 12,846 | 276 | 8.76 kDa | pH 5.73 | hypothetical protein PJG4_024 [Pseudomonas phage JG004] | hypothetical protein CDS | [YP_007002504.1](http://www.ncbi.nlm.nih.gov/protein/418488251?report=genbank&log$=prottop&blast_rank=1&RID=4VVW0JVV015) | 2.00E-60 | |
| EPA1_0028 | ← | 12,848 | 13,084 | 237 | 31.77 kDa | pH 4.92 | hypothetical protein K8_029 [Pseudomonas phage K8] | hypothetical protein CDS | [ALF51351.1](http://www.ncbi.nlm.nih.gov/protein/930348904?report=genbank&log$=prottop&blast_rank=1&RID=4VVW0JVV015) | 8.00E-49 | |
| EPA1_0029 | ← | 13,096 | 13,650 | 555 | 15.83 kDa | pH 6.79 | putative phosphoesterase [Pseudomonas phage vB_PaeM_C2-10_Ab1] | phosphoesterase | [YP_007236845.1](http://www.ncbi.nlm.nih.gov/protein/431809957?report=genbank&log$=prottop&blast_rank=1&RID=4VVW0JVV015) | 2.00E-133 | |
| EPA1_0030 | ← | 13,650 | 14,081 | 432 | 34.71 kDa | pH 5.74 | hypothetical protein PJG4_027 [Pseudomonas phage JG004] | hypothetical protein CDS | [YP_007002501.1](http://www.ncbi.nlm.nih.gov/protein/418488254?report=genbank&log$=prottop&blast_rank=1&RID=4VVW0JVV015) | 4.00E-99 | |
| EPA1_0031 | ← | 14,071 | 14,631 | 561 | 15.19 kDa | pH 10.11 | putative phosphohydrolase [Pseudomonas phage JG004] | phosphohydrolase | [YP_007002500.1](http://www.ncbi.nlm.nih.gov/protein/418488255?report=genbank&log$=prottop&blast_rank=1&RID=4VVW0JVV015) | 5.00E-136 | |
| EPA1_0032 | ← | 14,633 | 15,193 | 561 | 10.42 kDa | pH 6.68 | putative cell wall hydrolase [Pseudomonas phage JG004] | cell wall hydrolase | [YP_007002499.1](http://www.ncbi.nlm.nih.gov/protein/418488256?report=genbank&log$=prottop&blast_rank=1&RID=4VVW0JVV015) | 3.00E-136 | |
| EPA1_0033 | ← | 15,251 | 15,715 | 465 | 8.70 kDa | pH 4.23 | hypothetical protein PJG4_030 [Pseudomonas phage JG004] | hypothetical protein CDS | [YP_007002498.1](http://www.ncbi.nlm.nih.gov/protein/418488257?report=genbank&log$=prottop&blast_rank=1&RID=4VVW0JVV015) | 1.00E-111 | |
| EPA1_0034 | ← | 15,728 | 16,936 | 1,209 | 21.93 kDa | pH 6.84 | putative DNA ligase [Pseudomonas phage PAK_P2] | DNA ligase | [YP_008857197.1](http://www.ncbi.nlm.nih.gov/protein/563397432?report=genbank&log$=prottop&blast_rank=1&RID=4VVW0JVV015) | 0 | |
| EPA1_0035 | ← | 16,933 | 17,349 | 417 | 17.06 kDa | pH 5.34 | [Pseudomonas phage PAK_P1] | deoxycytidylate deaminase CDS | [YP_004327179.1](http://www.ncbi.nlm.nih.gov/protein/326804375?report=genbank&log$=prottop&blast_rank=1&RID=4VVW0JVV015) | 2.00E-95 | |
| EPA1_0036 | ← | 17,352 | 17,588 | 237 | 21.08 kDa | pH 5.95 | hypothetical protein MULTISPECIES: hypothetical protein [Delftia] | hypothetical protein CDS | [WP_016064767.1](http://www.ncbi.nlm.nih.gov/protein/506600325?report=genbank&log$=prottop&blast_rank=1&RID=5VVGE2DR014) | 3.00E-48 | |
| EPA1_0037 | ← | 17,598 | 17,810 | 213 | 21.47 kDa | pH 9.53 | hypothetical protein PAK_P100165c [Pseudomonas phage PAK_P1] | hypothetical protein CDS | [YP_004327181.1](http://www.ncbi.nlm.nih.gov/protein/326804377?report=genbank&log$=prottop&blast_rank=1&RID=5VVGE2DR014) | 2.00E-41 | |
| EPA1_0038 | ← | 17,807 | 18,076 | 270 | 17.31 kDa | pH 9.39 | hypothetical protein MULTISPECIES: hypothetical protein [Delftia] | hypothetical protein CDS | [WP_016057384.1](http://www.ncbi.nlm.nih.gov/protein/506591628?report=genbank&log$=prottop&blast_rank=1&RID=5VVGE2DR014) | 3.00E-57 | |
| EPA1_0039 | ← | 18,085 | 18,744 | 660 | 46.28 kDa | pH 5.62 | hypothetical protein PAK_P200161c [Pseudomonas phage PAK_P2] | hypothetical protein CDS | [YP_008857202.1](http://www.ncbi.nlm.nih.gov/protein/563397437?report=genbank&log$=prottop&blast_rank=1&RID=5VVGE2DR014) | 9.00E-162 | |
| EPA1_0040 | ← | 18,746 | 19,093 | 348 | 15.45 kDa | pH 7.50 | hypothetical protein PAK_P200162c [Pseudomonas phage PAK_P2] | hypothetical protein CDS | [YP_008857203.1](http://www.ncbi.nlm.nih.gov/protein/563397438?report=genbank&log$=prottop&blast_rank=1&RID=5VVGE2DR014) | 1.00E-78 | |
| EPA1_0041 | ← | 19,074 | 19,352 | 279 | 8.93 kDa | pH 3.62 | hypothetical protein K8_041 [Pseudomonas phage K8] | hypothetical protein CDS | [ALF51363.1](http://www.ncbi.nlm.nih.gov/protein/930348916?report=genbank&log$=prottop&blast_rank=1&RID=5VVGE2DR014) | 7.00E-56 | |
| EPA1_0042 | ← | 19,662 | 19,847 | 186 | 7.45 kDa | pH 6.51 | hypothetical protein C11_040 [Pseudomonas phage C11] | hypothetical protein CDS | [ALJ97500.1](http://www.ncbi.nlm.nih.gov/protein/940162608?report=genbank&log$=prottop&blast_rank=1&RID=5VVGE2DR014) | 2.00E-60 | |
| EPA1_0043 | ← | 19,349 | 19,627 | 279 | 10.00 kDa | pH 4.33 | MULTISPECIES: hypothetical protein [Proteobacteria] | hypothetical protein CDS | [WP_016064770.1](http://www.ncbi.nlm.nih.gov/protein/506600328?report=genbank&log$=prottop&blast_rank=1&RID=5VVGE2DR014) | 4.00E-37 | |
| EPA1_0044 | ← | 19,848 | 20,141 | 294 | 25.39 kDa | pH 9.58 | hypothetical protein BN405_2-10_Ab1_orf_38 [Pseudomonas phage vB_PaeM_C2-10_Ab1] | hypothetical protein CDS | [YP_007236859.1](http://www.ncbi.nlm.nih.gov/protein/431809971?report=genbank&log$=prottop&blast_rank=1&RID=5VVGE2DR014) | 7.00E-66 | |
| EPA1_0045 | ← | 20,138 | 20,323 | 186 | 13.10 kDa | pH 10.06 | hypothetical protein PJG4_041 [Pseudomonas phage JG004] | hypothetical protein CDS | [YP_007002487.1](http://www.ncbi.nlm.nih.gov/protein/418488268?report=genbank&log$=prottop&blast_rank=1&RID=5VVGE2DR014) | 5.00E-34 | |
| EPA1_0046 | ← | 20,384 | 20,932 | 549 | 10.56 kDa | pH 3.73 | putative protease subunit [Pseudomonas phage JG004] | protease subunit | [YP_007002486.1](http://www.ncbi.nlm.nih.gov/protein/418488269?report=genbank&log$=prottop&blast_rank=1&RID=5VVGE2DR014) | 7.00E-131 | |
| EPA1_0047 | ← | 20,980 | 21,336 | 357 | 10.49 kDa | pH 4.08 | hypothetical protein PJG4_043 [Pseudomonas phage JG004] | hypothetical protein CDS | [YP_007002485.1](http://www.ncbi.nlm.nih.gov/protein/418488270?report=genbank&log$=prottop&blast_rank=1&RID=5VVGE2DR014) | 1.00E-76 | |
| EPA1_0048 | ← | 21,333 | 21,800 | 468 | 7.12 kDa | pH 4.79 | hypothetical protein PJG4_044 [Pseudomonas phage JG004] | hypothetical protein CDS | [YP_007002484.1](http://www.ncbi.nlm.nih.gov/protein/418488271?report=genbank&log$=prottop&blast_rank=1&RID=5VVGE2DR014) | 5.00E-109 | |
| EPA1_0048A | → | 22,274 | 22,462 | 189 | 11.41 kDa | pH 6.63 | hypothetical protein PaP1_gp160 [Pseudomonas phage PaP1] | hypothetical protein CDS | [YP_009047069.1](http://www.ncbi.nlm.nih.gov/protein/663463633?report=genbank&log$=prottop&blast_rank=1&RID=5VVGE2DR014) | 1.00E-16 | |
| EPA1_0049 | → | 22,605 | 22,928 | 324 | 7.16 kDa | pH 3.92 | hypothetical protein PAK_P100177 [Pseudomonas phage PAK_P1] | hypothetical protein CDS | [YP_004327192.1](http://www.ncbi.nlm.nih.gov/protein/326804388?report=genbank&log$=prottop&blast_rank=1&RID=5VVGE2DR014) | 5.00E-70 | |
| EPA1_0050 | → | 23,093 | 23,227 | 135 | 20.40 kDa | pH 4.17 | hypothetical protein PAK_P200173 [Pseudomonas phage PAK_P2] | hypothetical protein CDS | [YP_008857214.1](http://www.ncbi.nlm.nih.gov/protein/563397449?report=genbank&log$=prottop&blast_rank=1&RID=5VVGE2DR014) | 1.00E-19 | |
| EPA1_0051 | ← | 24,221 | 24,337 | 117 | 18.07 kDa | pH 4.75 | hypothetical protein PAK_P100180c [Pseudomonas phage PAK_P1] | hypothetical protein CDS | [YP_008869212.1](http://www.ncbi.nlm.nih.gov/protein/563448594?report=genbank&log$=prottop&blast_rank=1&RID=5VVNKXXD015) | 2.00E-16 | |
| EPA1_0052 | ← | 24,698 | 24,814 | 117 | 4.00 kDa | pH 8.04 | protein of unknown function [Nitrospira sp. ENR4] | hypothetical protein CDS | [CUQ66600.1](http://www.ncbi.nlm.nih.gov/protein/952975679?report=genbank&log$=prottop&blast_rank=1&RID=5VVNKXXD015) | 0.029 | |
| EPA1_0053 | ← | 25,137 | 25,448 | 312 | 12.03 kDa | pH 6.93 | MULTISPECIES: hypothetical protein [Delftia] | hypothetical protein CDS | [WP_016057394.1](http://www.ncbi.nlm.nih.gov/protein/506591640?report=genbank&log$=prottop&blast_rank=1&RID=5VVNKXXD015) | 2.00E-10 | |
| EPA1_0054 | → | 25,648 | 27,168 | 1,521 | 5.02 kDa | pH 6.45 | terminase large subunit [Pseudomonas phage JG004] | Phage terminase, large subunit CDS | [YP_007002481.1](http://www.ncbi.nlm.nih.gov/protein/418488274?report=genbank&log$=prottop&blast_rank=1&RID=5VVNKXXD015) | 0.0 | |
| EPA1_0055 | → | 27,181 | 28,620 | 1,440 | 6.75 kDa | pH 6.75 | MULTISPECIES: hypothetical protein [Delftia] | hypothetical protein CDS | [WP_016057396.1](http://www.ncbi.nlm.nih.gov/protein/506591642?report=genbank&log$=prottop&blast_rank=1&RID=5VVNKXXD015) | 0.0 | |
| EPA1_0056 | → | 28,630 | 29,100 | 471 | 4.00 kDa | pH 6.43 | putative methyltransferase [Pseudomonas phage JG004] | methyltransferase | [YP_007002479.1](http://www.ncbi.nlm.nih.gov/protein/418488276?report=genbank&log$=prottop&blast_rank=1&RID=5VVNKXXD015) | 1.00E-108 | |
| EPA1_0057 | → | 29,097 | 30,014 | 918 | 4.34 kDa | pH 8.49 | hypothetical protein PJG4_062 [Pseudomonas phage JG004] | hypothetical protein CDS | [YP_007002478.1](http://www.ncbi.nlm.nih.gov/protein/418488277?report=genbank&log$=prottop&blast_rank=1&RID=5VVNKXXD015) | 0.0 | |
| EPA1_0058 | → | 30,042 | 30,452 | 411 | 11.44 kDa | pH 7.63 | hypothetical protein PJG4_063 [Pseudomonas phage JG004] | hypothetical protein CDS | [YP_007002477.1](http://www.ncbi.nlm.nih.gov/protein/418488278?report=genbank&log$=prottop&blast_rank=1&RID=5VVNKXXD015) | 1.00E-92 | |
| EPA1_0059 | → | 30,496 | 31,530 | 1,035 | 57.09 kDa | pH 6.10 | MULTISPECIES: phage capsid protein [Delftia] | major capsid protein CDS | [WP_016037448.1](http://www.ncbi.nlm.nih.gov/protein/506571222?report=genbank&log$=prottop&blast_rank=1&RID=5VVNKXXD015) | 0.0 | |
| EPA1_0060 | → | 31,583 | 32,059 | 477 | 54.23 kDa | pH 5.35 | putative methyltransferase [Pseudomonas phage JG004] | methyltransferase | [YP_007002479.1](http://www.ncbi.nlm.nih.gov/protein/418488276?report=genbank&log$=prottop&blast_rank=1&RID=5VVNKXXD015) | 1.00E-108 | |
| EPA1_0061 | → | 32,097 | 32,510 | 414 | 17.16 kDa | pH 4.06 | hypothetical protein PAK_P400008 [Pseudomonas phage PAK_P4] | phage tail CDS | [YP_008859219.1](http://www.ncbi.nlm.nih.gov/protein/563399378?report=genbank&log$=prottop&blast_rank=1&RID=5VVNKXXD015) | 3.00E-97 | |
| EPA1_0062 | → | 32,510 | 32,890 | 381 | 33.07 kDa | pH 4.56 | hypothetical protein PJG4_067 [Pseudomonas phage JG004] | hypothetical protein CDS | [YP_007002473.1](http://www.ncbi.nlm.nih.gov/protein/418488282?report=genbank&log$=prottop&blast_rank=1&RID=5VVNKXXD015) | 1.00E-88 | |
| EPA1_0063 | → | 32,887 | 33,450 | 564 | 14.85 kDa | pH 5.59 | hypothetical protein BN405_2-10_Ab1_orf_56 [Pseudomonas phage vB_PaeM_C2-10_Ab1] | hypothetical protein CDS | [YP_007236877.1](http://www.ncbi.nlm.nih.gov/protein/431809989?report=genbank&log$=prottop&blast_rank=1&RID=5VVNKXXD015) | 2.00E-137 | |
| EPA1_0064 | → | 33,463 | 34,749 | 1,287 | 39.38 kDa | pH 5.16 | hypothetical protein PAK_P200011 [Pseudomonas phage PAK_P2] | hypothetical protein CDS | [YP_008857051.1](http://www.ncbi.nlm.nih.gov/protein/563397286?report=genbank&log$=prottop&blast_rank=1&RID=5VVNKXXD015) | 0.0 | |
| EPA1_0065 | → | 34,780 | 35,304 | 525 | 18.16 kDa | pH 4.49 | hypothetical protein PAK_P100012 [Pseudomonas phage PAK_P1] | hypothetical protein CDS | [YP_004327205.1](http://www.ncbi.nlm.nih.gov/protein/326804401?report=genbank&log$=prottop&blast_rank=1&RID=5VVNKXXD015) | 3.00E-123 | |
| EPA1_0066 | → | 35,379 | 35,879 | 501 | 15.65 kDa | pH 6.81 | hypothetical protein BN405_2-10_Ab1_orf_59 [Pseudomonas phage vB_PaeM_C2-10_Ab1] | hypothetical protein CDS | [YP_007236880.1](http://www.ncbi.nlm.nih.gov/protein/431809992?report=genbank&log$=prottop&blast_rank=1&RID=5VVNKXXD015) | 1.00E-117 | |
| EPA1_0067 | → | 35,879 | 36,358 | 480 | 14.35 kDa | pH 8.19 | MULTISPECIES: hypothetical protein [Delftia] | hypothetical protein CDS | [WP_016057407.1](http://www.ncbi.nlm.nih.gov/protein/506591655?report=genbank&log$=prottop&blast_rank=1&RID=5VVNKXXD015) | 1.00E-111 | |
| EPA1_0068 | → | 36,372 | 36,743 | 372 | 21.23 kDa | pH 5.25 | MULTISPECIES: hypothetical protein [Delftia] | hypothetical protein CDS | [WP_016057408.1](http://www.ncbi.nlm.nih.gov/protein/506591656?report=genbank&log$=prottop&blast_rank=1&RID=5VVNKXXD015) | 3.00E-83 | |
| EPA1_0069 | → | 36,851 | 37,003 | 153 | 46.36 kDa | pH 4.72 | hypothetical protein PAK_P200016 [Pseudomonas phage PAK_P2] | hypothetical protein CDS | [YP_008857056.1](http://www.ncbi.nlm.nih.gov/protein/563397291?report=genbank&log$=prottop&blast_rank=1&RID=5VVNKXXD015) | 1.00E-25 | |
| EPA1_0070 | → | 37,000 | 39,366 | 2,367 | 18.25 kDa | pH 5.18 | putative tape measure protein [Pseudomonas phage vB_PaeM_C2-10_Ab1] | tape measure protein | [YP_007236883.1](http://www.ncbi.nlm.nih.gov/protein/431809995?report=genbank&log$=prottop&blast_rank=1&RID=5VVNKXXD015) | 0.0 | |
| EPA1_0071 | → | 39,363 | 40,124 | 762 | 17.70 kDa | pH 6.24 | hypothetical protein [Pseudomonas phage vB_PaeM_C2-10_Ab02] | hypothetical protein CDS | [CEF88998.1](http://www.ncbi.nlm.nih.gov/protein/713662156?report=genbank&log$=prottop&blast_rank=1&RID=5VVNKXXD015) | 0.0 | |
| EPA1_0072 | → | 40,130 | 40,486 | 357 | 13.62 kDa | pH 4.75 | hypothetical protein BN405_2-10_Ab1_orf_65 [Pseudomonas phage vB_PaeM_C2-10_Ab1] | hypothetical protein CDS | [YP_007236886.1](http://www.ncbi.nlm.nih.gov/protein/431809998?report=genbank&log$=prottop&blast_rank=1&RID=5VVNKXXD015) | 7.00E-81 | |
| EPA1_0073 | → | 40,483 | 41,400 | 918 | 5.61 kDa | pH 4.58 | hypothetical protein PAK_P400021 [Pseudomonas phage PAK_P4] | hypothetical protein CDS | [YP_008859232.1](http://www.ncbi.nlm.nih.gov/protein/563399391?report=genbank&log$=prottop&blast_rank=1&RID=5VVNKXXD015) | 0.0 | |
| EPA1_0074 | → | 41,397 | 42,137 | 741 | 85.93 kDa | pH 9.28 | putative baseplate protein [Pseudomonas phage PaP1] | baseplate protein | [YP_007236476.1](http://www.ncbi.nlm.nih.gov/protein/431809583?report=genbank&log$=prottop&blast_rank=1&RID=5VVNKXXD015) | 0.0 | |
| EPA1_0075 | → | 42,148 | 42,519 | 372 | 26.69 kDa | pH 7.72 | hypothetical protein BN405_2-10_Ab1_orf_68 [Pseudomonas phage vB_PaeM_C2-10_Ab1] | baseplate wedge protein CDS | [YP_007236889.1](http://www.ncbi.nlm.nih.gov/protein/431810001?report=genbank&log$=prottop&blast_rank=1&RID=5VVNKXXD015) | 4.00E-84 | |
| EPA1_0076 | → | 42,521 | 43,984 | 1,464 | 14.18 kDa | pH 4.55 | Putative baseplate component [Pseudomonas phage vB_PaeM_C2-10_Ab1] | baseplate component | [YP_007236890.1](http://www.ncbi.nlm.nih.gov/protein/431810002?report=genbank&log$=prottop&blast_rank=1&RID=5VVNWRN7014) | 0.0 | |
| EPA1_0077 | → | 44,003 | 44,734 | 732 | 52.35 kDa | pH 4.31 | hypothetical protein BN405_2-10_Ab1_orf_70 [Pseudomonas phage vB_PaeM_C2-10_Ab1] | hypothetical protein CDS | [YP_007236891.1](http://www.ncbi.nlm.nih.gov/protein/431810003?report=genbank&log$=prottop&blast_rank=1&RID=5VVNWRN7014) | 1.00E-172 | |
| EPA1_0078 | → | 44,745 | 46,802 | 2,058 | 26.66 kDa | pH 4.50 | putative tail fiber protein [Pseudomonas phage C11] | Phage tail fiber protein | [ALJ97533.1](http://www.ncbi.nlm.nih.gov/protein/940162641?report=genbank&log$=prottop&blast_rank=1&RID=5VVNWRN7014) | 0.0 | |
| EPA1_0079 | → | 46,846 | 47,220 | 375 | 71.86 kDa | pH 9.22 | hypothetical protein PJG4_085 [Pseudomonas phage JG004] | tail fibre assembly protein CDS | [YP_007002455.1](http://www.ncbi.nlm.nih.gov/protein/418488300?report=genbank&log$=prottop&blast_rank=1&RID=5VVNWRN7014) | 5.00E-84 | |
| EPA1_0080 | → | 47,234 | 48,733 | 1,500 | 14.54 kDa | pH 5.12 | putative tail fiber protein [Pseudomonas phage vB_PaeM_C2-10_Ab02] | Phage tail fiber protein CDS | [CEF89007.1](http://www.ncbi.nlm.nih.gov/protein/713662165?report=genbank&log$=prottop&blast_rank=1&RID=5VVNWRN7014) | 0.0 | |
| EPA1_0081 | → | 48,750 | 49,310 | 561 | 53.07 kDa | pH 7.65 | Putative peptidoglycan binding protein [Pseudomonas phage vB_PaeM_C2-10_Ab02] | phage-encoded peptidoglycan binding protein CDS | [CEF89008.1](http://www.ncbi.nlm.nih.gov/protein/713662166?report=genbank&log$=prottop&blast_rank=2&RID=5W8EG66K015) | 1.00E-134 | |
| EPA1_0082 | → | 49,328 | 49,567 | 240 | 20.93 kDa | pH 9.68 | hypothetical protein BN405_2-10_Ab1_orf_75 [Pseudomonas phage vB_PaeM_C2-10_Ab1] | hypothetical protein CDS | [YP_007236896.1](http://www.ncbi.nlm.nih.gov/protein/431810008?report=genbank&log$=prottop&blast_rank=1&RID=5VVNWRN7014) | 3.00E-47 | |
| EPA1_0083 | → | 49,554 | 49,997 | 444 | 8.46 kDa | pH 4.62 | hypothetical protein BN405_2-10_Ab1_orf_76 [Pseudomonas phage vB_PaeM_C2-10_Ab1] | hypothetical protein CDS | [YP_007236897.1](http://www.ncbi.nlm.nih.gov/protein/431810009?report=genbank&log$=prottop&blast_rank=1&RID=5VVNWRN7014) | 3.00E-103 | |
| EPA1_0084 | → | 50,008 | 50,139 | 132 | 15.89 kDa | pH 4.62 | hypothetical protein PAK_P200031 [Pseudomonas phage PAK_P2] | hypothetical protein CDS | [YP_008857071.1](http://www.ncbi.nlm.nih.gov/protein/563397306?report=genbank&log$=prottop&blast_rank=1&RID=5VVNWRN7014) | 3.00E-22 | |
| EPA1_0085 | → | 50,129 | 50,434 | 306 | 4.86 kDa | pH 8.49 | MULTISPECIES: hypothetical protein [Delftia] | hypothetical protein CDS | [WP_016057425.1](http://www.ncbi.nlm.nih.gov/protein/506591677?report=genbank&log$=prottop&blast_rank=1&RID=5VVNWRN7014) | 4.00E-64 | |
| EPA1_0086 | → | 50,470 | 50,784 | 315 | 11.47 kDa | pH 6.90 | hypothetical protein PAK_P200033 [Pseudomonas phage PAK_P2] | hypothetical protein CDS | [YP_008857073.1](http://www.ncbi.nlm.nih.gov/protein/563397308?report=genbank&log$=prottop&blast_rank=1&RID=5VVNWRN7014) | 2.00E-67 | |
| EPA1_0087 | ← | 50,832 | 51,140 | 309 | 12.36 kDa | pH 9.08 | hypothetical protein BN405_2-10_Ab1_orf_78 [Pseudomonas phage vB_PaeM_C2-10_Ab1] | hypothetical protein CDS | [YP_007236899.1](http://www.ncbi.nlm.nih.gov/protein/431810011?report=genbank&log$=prottop&blast_rank=1&RID=5VVNWRN7014) | 3.00E-66 | |
| EPA1_0088 | ← | 51,152 | 51,472 | 321 | 11.46 kDa | pH 6.53 | hypothetical protein BN405_2-10_Ab1_orf_79 [Pseudomonas phage vB_PaeM_C2-10_Ab1] | hypothetical protein CDS | [YP_007236900.1](http://www.ncbi.nlm.nih.gov/protein/431810012?report=genbank&log$=prottop&blast_rank=1&RID=5VVNWRN7014) | 3.00E-72 | |
| EPA1_0089 | ← | 51,474 | 52,283 | 810 | 12.13 kDa | pH 5.44 | hypothetical protein BN405_2-10_Ab1_orf_79 [Pseudomonas phage vB_PaeM_C2-10_Ab1] | Nucleoside triphosphate pyrophosphohydrolase CDS | [YP_007236900.1](http://www.ncbi.nlm.nih.gov/protein/431810012?report=genbank&log$=prottop&blast_rank=1&RID=5VVNWRN7014) | 3.00E-72 | |
| EPA1_0090 | ← | 52,276 | 52,431 | 156 | 30.56 kDa | pH 4.59 | hypothetical protein PJG4_095 [Pseudomonas phage JG004] | hypothetical protein CDS | [YP_007002445.1](http://www.ncbi.nlm.nih.gov/protein/418488310?report=genbank&log$=prottop&blast_rank=1&RID=5VVNWRN7014) | 3.00E-27 | |
| EPA1_0091 | ← | 52,434 | 53,579 | 1,146 | 5.75 kDa | pH 3.21 | hypothetical protein [Pseudomonas phage vB_PaeM_C2-10_Ab02] | RNA ligase CDS | [CEF89017.1](http://www.ncbi.nlm.nih.gov/protein/713662175?report=genbank&log$=prottop&blast_rank=1&RID=5VVNWRN7014) | 0.0 | |
| EPA1_0092 | ← | 53,611 | 53,844 | 234 | 43.00 kDa | pH 5.39 | hypothetical protein PJG4_097 [Pseudomonas phage JG004] | hypothetical protein CDS | [YP_007002443.1](http://www.ncbi.nlm.nih.gov/protein/418488312?report=genbank&log$=prottop&blast_rank=1&RID=5VVNWRN7014) | 1.00E-47 | |
| EPA1_0093 | ← | 53,882 | 54,115 | 234 | 8.64 kDa | pH 7.54 | MULTISPECIES: hypothetical protein [Delftia] | hypothetical protein CDS | [WP_016057432.1](http://www.ncbi.nlm.nih.gov/protein/506591685?report=genbank&log$=prottop&blast_rank=1&RID=5VVNWRN7014) | 6.00E-47 | |
| EPA1_0093A | ← | 54,404 | 54,511 | 108 | 12.89 kDa | pH 3.62 | hypothetical protein BN405_2-10_Ab1_orf_83 [Pseudomonas phage vB_PaeM_C2-10_Ab1] | hypothetical protein CDS | [YP_007236904.1](http://www.ncbi.nlm.nih.gov/protein/431810016?report=genbank&log$=prottop&blast_rank=1&RID=HYXX01Z2014) | 3.00E-12 | |
| EPA1_0094 | → | 54,568 | 54,765 | 198 | 8.53 kDa | pH 4.07 | hypothetical protein BN405_2-10_Ab1_orf_84 [Pseudomonas phage vB_PaeM_C2-10_Ab1] | hypothetical protein CDS | [YP_007236905.1](http://www.ncbi.nlm.nih.gov/protein/431810017?report=genbank&log$=prottop&blast_rank=1&RID=5VVNWRN7014) | 1.00E-38 | |
| EPA1_0095 | → | 54,768 | 55,253 | 486 | 7.47 kDa | pH 3.90 | hypothetical protein PAK_P200042 [Pseudomonas phage PAK_P2] | hypothetical protein CDS | [YP_008857082.1](http://www.ncbi.nlm.nih.gov/protein/563397317?report=genbank&log$=prottop&blast_rank=1&RID=5VVNWRN7014) | 2.00E-116 | |
| EPA1_0096 | → | 55,281 | 55,529 | 249 | 19.12 kDa | pH 9.53 | hypothetical protein K8_093 [Pseudomonas phage K8] | hypothetical protein CDS | [ALF51414.1](http://www.ncbi.nlm.nih.gov/protein/930348967?report=genbank&log$=prottop&blast_rank=1&RID=5VVNWRN7014) | 9.00E-52 | |
| EPA1_0097 | → | 55,531 | 55,920 | 390 | 9.26 kDa | pH 6.24 | hypothetical protein [Pseudomonas phage vB_PaeM_C2-10_Ab02] | hypothetical protein CDS | [CEF89022.1](http://www.ncbi.nlm.nih.gov/protein/713662180?report=genbank&log$=prottop&blast_rank=1&RID=5VVNWRN7014) | 3.00E-88 | |
| EPA1_0098 | → | 55,917 | 56,582 | 666 | 14.67 kDa | pH 4.66 | hypothetical protein PAK_P100045 [Pseudomonas phage PAK_P1] | hypothetical protein CDS | [YP_004327236.1](http://www.ncbi.nlm.nih.gov/protein/326804432?report=genbank&log$=prottop&blast_rank=1&RID=5VVNWRN7014) | 4.00E-158 | |
| EPA1_0099 | → | 56,569 | 56,733 | 165 | 24.51 kDa | pH 4.42 | hypothetical protein BN405_2-10_Ab1_orf_90 [Pseudomonas phage vB_PaeM_C2-10_Ab1] | hypothetical protein CDS | [YP_007236911.1](http://www.ncbi.nlm.nih.gov/protein/431810023?report=genbank&log$=prottop&blast_rank=1&RID=5VVNWRN7014) | 2.00E-30 | |
| EPA1_0100 | → | 56,736 | 57,038 | 303 | 6.43 kDa | pH 3.87 | hypothetical protein [Pseudomonas phage vB_PaeM_C2-10_Ab02] | hypothetical protein CDS | [CEF89025.1](http://www.ncbi.nlm.nih.gov/protein/713662183?report=genbank&log$=prottop&blast_rank=1&RID=5VVNWRN7014) | 1.00E-66 | |
| EPA1_0101 | → | 57,039 | 57,461 | 423 | 11.95 kDa | pH 4.17 | hypothetical protein PAK_P100048 [Pseudomonas phage PAK_P1] | hypothetical protein CDS | [YP_004327239.1](http://www.ncbi.nlm.nih.gov/protein/326804435?report=genbank&log$=prottop&blast_rank=1&RID=5VVP0YEY015) | 2.00E-100 | |
| EPA1_0102 | → | 57,579 | 57,707 | 129 | 16.49 kDa | pH 8.67 | hypothetical protein PAK_P100050 [Pseudomonas phage PAK_P1] | hypothetical protein CDS | [YP_008869167.1](http://www.ncbi.nlm.nih.gov/protein/563448549?report=genbank&log$=prottop&blast_rank=1&RID=5VVP0YEY015) | 3.00E-20 | |
| EPA1_0103 | → | 57,694 | 57,885 | 192 | 5.10 kDa | pH 11.15 | hypothetical protein PAK_P100051 [Pseudomonas phage PAK_P1] | hypothetical protein CDS | [YP_004327241.1](http://www.ncbi.nlm.nih.gov/protein/326804437?report=genbank&log$=prottop&blast_rank=1&RID=5VVP0YEY015) | 2.00E-36 | |
| EPA1_0104 | → | 57,895 | 58,140 | 246 | 7.12 kDa | pH 4.21 | hypothetical protein PAK_P100052 [Pseudomonas phage PAK_P1] | hypothetical protein CDS | [YP_008869168.1](http://www.ncbi.nlm.nih.gov/protein/563448550?report=genbank&log$=prottop&blast_rank=1&RID=5VVP0YEY015) | 2.00E-53 | |
| EPA1_0105 | → | 58,137 | 58,322 | 186 | 9.25 kDa | pH 4.18 | hypothetical protein PAK_P100053 [Pseudomonas phage PAK_P1] | hypothetical protein CDS | [YP_008869169.1](http://www.ncbi.nlm.nih.gov/protein/563448551?report=genbank&log$=prottop&blast_rank=1&RID=5VVP0YEY015) | 1.00E-37 | |
| EPA1_0106 | → | 58,376 | 60,238 | 1,863 | 7.17 kDa | pH 5.56 | MULTISPECIES: DNA primase/helicase [Delftia] | DNA primase/helicase | [WP_016057444.1](http://www.ncbi.nlm.nih.gov/protein/506591699?report=genbank&log$=prottop&blast_rank=1&RID=5VVP0YEY015) | 0.0 | |
| EPA1_0107 | → | 60,299 | 63,013 | 2,715 | 70.57 kDa | pH 4.96 | putative DNA Polymerase [Pseudomonas phage vB_PaeM_C2-10_Ab08] | DNA Polymerase | [CEF89418.1](http://www.ncbi.nlm.nih.gov/protein/713662610?report=genbank&log$=prottop&blast_rank=1&RID=5VVP0YEY015) | 0.0 | |
| EPA1_0108 | → | 63,104 | 63,502 | 399 | 103.44 kDa | pH 6.63 | hypothetical protein PAK_P400055 [Pseudomonas phage PAK_P4] | hypothetical protein CDS | [YP_008859267.1](http://www.ncbi.nlm.nih.gov/protein/563399426?report=genbank&log$=prottop&blast_rank=1&RID=5VVP0YEY015) | 2.00E-84 | |
| EPA1_0109 | → | 63,532 | 63,699 | 168 | 14.28 kDa | pH 8.92 | hypothetical protein PaP1_gp092 [Pseudomonas phage PaP1] | hypothetical protein CDS | [YP_007236503.1](http://www.ncbi.nlm.nih.gov/protein/431809610?report=genbank&log$=prottop&blast_rank=1&RID=5VVP0YEY015) | 6.00E-31 | |
| EPA1_0110 | → | 63,701 | 64,405 | 705 | 6.15 kDa | pH 10.30 | MULTISPECIES: hypothetical protein [Delftia] | hypothetical protein CDS | [WP_016064808.1](http://www.ncbi.nlm.nih.gov/protein/506600368?report=genbank&log$=prottop&blast_rank=1&RID=5VVP0YEY015) | 9.00E-148 | |
| EPA1_0111 | → | 64,507 | 65,511 | 1,005 | 26.06 kDa | pH 4.70 | hypothetical protein BN405_2-10_Ab1_orf_101 [Pseudomonas phage vB_PaeM_C2-10_Ab1] | hypothetical protein CDS | [YP_007236922.1](http://www.ncbi.nlm.nih.gov/protein/431810034?report=genbank&log$=prottop&blast_rank=1&RID=5VVP0YEY015) | 0.0 | |
| EPA1_0112 | → | 65,591 | 65,842 | 252 | 37.13 kDa | pH 4.98 | hypothetical protein BN405_2-10_Ab1_orf_103 [Pseudomonas phage vB_PaeM_C2-10_Ab1] | hypothetical protein CDS | [YP_007236924.1](http://www.ncbi.nlm.nih.gov/protein/431810036?report=genbank&log$=prottop&blast_rank=1&RID=5VVP0YEY015) | 7.00E-49 | |
| EPA1_0113 | → | 65,849 | 66,073 | 225 | 9.22 kDa | pH 10.87 | hypothetical protein BN405_2-10_Ab1_orf_104 [Pseudomonas phage vB_PaeM_C2-10_Ab1] | hypothetical protein CDS | [YP_007236925.1](http://www.ncbi.nlm.nih.gov/protein/431810037?report=genbank&log$=prottop&blast_rank=1&RID=5VVP0YEY015) | 1.00E-45 | |
| EPA1_0114 | → | 66,115 | 67,167 | 1,053 | 8.51 kDa | pH 4.72 | Putative exodeoxyribonuclease [Pseudomonas phage vB_PaeM_C2-10_Ab1] | exodeoxyribonuclease | [YP_007236926.1](http://www.ncbi.nlm.nih.gov/protein/431810038?report=genbank&log$=prottop&blast_rank=1&RID=5VVP0YEY015) | 0.0 | |
| EPA1_0115 | → | 67,164 | 67,727 | 564 | 40.07 kDa | pH 4.95 | phage protein [Pseudomonas phage vB_PaeM_C2-10_Ab1] | HNH endonuclease CDS | [YP_007236927.1](http://www.ncbi.nlm.nih.gov/protein/431810039?report=genbank&log$=prottop&blast_rank=1&RID=5VVP0YEY015) | 2.00E-132 | |
| EPA1_0116 | → | 67,724 | 68,122 | 399 | 21.51 kDa | pH 9.71 | hypothetical protein PaP1_gp099 [Pseudomonas phage PaP1] | hypothetical protein CDS | [YP_007236510.1](http://www.ncbi.nlm.nih.gov/protein/431809617?report=genbank&log$=prottop&blast_rank=1&RID=5VVP0YEY015) | 3.00E-92 | |
| EPA1_0117 | → | 68,119 | 68,349 | 231 | 15.19 kDa | pH 8.67 | hypothetical protein PAK_P200064 [Pseudomonas phage PAK_P2] | hypothetical protein CDS | [YP_008857105.1](http://www.ncbi.nlm.nih.gov/protein/563397340?report=genbank&log$=prottop&blast_rank=1&RID=5VVP0YEY015) | 2.00E-44 | |
| EPA1_0118 | → | 68,346 | 68,783 | 438 | 8.65 kDa | pH 8.49 | hypothetical protein PAK_P200065 [Pseudomonas phage PAK_P2] | hypothetical protein CDS | [YP_008857106.1](http://www.ncbi.nlm.nih.gov/protein/563397341?report=genbank&log$=prottop&blast_rank=1&RID=5VVP0YEY015) | 1.00E-99 | |
| EPA1_0119 | → | 68,780 | 68,950 | 171 | 16.52 kDa | pH 9.64 | hypothetical protein PAK_P200066 [Pseudomonas phage PAK_P2] | hypothetical protein CDS | [YP_008857107.1](http://www.ncbi.nlm.nih.gov/protein/563397342?report=genbank&log$=prottop&blast_rank=1&RID=5VVP0YEY015) | 3.00E-31 | |
| EPA1_0120 | → | 68,952 | 69,134 | 183 | 6.56 kDa | pH 3.87 | hypothetical protein [Pseudomonas phage vB_PaeM_C2-10_Ab02] | hypothetical protein CDS | [CEF89047.1](http://www.ncbi.nlm.nih.gov/protein/713662205?report=genbank&log$=prottop&blast_rank=1&RID=5VVP0YEY015) | 1.00E-32 | |
| EPA1_0121 | → | 69,131 | 69,910 | 780 | 7.04 kDa | pH 3.68 | hypothetical protein PAK_P400068 [Pseudomonas phage PAK_P4] | metallophosphoesterase CDS | [YP_008859280.1](http://www.ncbi.nlm.nih.gov/protein/563399439?report=genbank&log$=prottop&blast_rank=1&RID=5VVP0YEY015) | 0.0 | |
| EPA1_0122 | → | 69,907 | 70,089 | 183 | 29.31 kDa | pH 7.75 | hypothetical protein PAK_P200069 [Pseudomonas phage PAK_P2] | hypothetical protein CDS | [YP_008857110.1](http://www.ncbi.nlm.nih.gov/protein/563397345?report=genbank&log$=prottop&blast_rank=1&RID=5VVP0YEY015) | 2.00E-34 | |
| EPA1_0123 | → | 70,101 | 70,310 | 210 | 6.90 kDa | pH 3.68 | hypothetical protein [Delftia sp. 670] | hypothetical protein CDS | [WP_016037507.1](http://www.ncbi.nlm.nih.gov/protein/506571281?report=genbank&log$=prottop&blast_rank=1&RID=5VVP0YEY015) | 2.00E-40 | |
| EPA1_0124 | → | 70,329 | 70,664 | 336 | 7.50 kDa | pH 4.58 | hypothetical protein PAK_P100072 [Pseudomonas phage PAK_P1] | hypothetical protein CDS | [YP_004327259.1](http://www.ncbi.nlm.nih.gov/protein/326804455?report=genbank&log$=prottop&blast_rank=1&RID=5VVP0YEY015) | 6.00E-73 | |
| EPA1_0125 | → | 70,668 | 70,880 | 213 | 12.44 kDa | pH 3.99 | hypothetical protein PJG4_128 [Pseudomonas phage JG004] | hypothetical protein CDS | [YP_007002412.1](http://www.ncbi.nlm.nih.gov/protein/418488343?report=genbank&log$=prottop&blast_rank=1&RID=5VVP0YEY015) | 9.00E-41 | |
| EPA1_0126 | → | 70,873 | 71,823 | 951 | 7.75 kDa | pH 3.98 | hypothetical protein PJG4_129 [Pseudomonas phage JG004] | hypothetical protein CDS | [YP_007002411.1](http://www.ncbi.nlm.nih.gov/protein/418488344?report=genbank&log$=prottop&blast_rank=1&RID=5VVPXKCM014) | 0.0 | |
| EPA1_0126A | → | 71,820 | 71,975 | 156 | 35.37 kDa | pH 4.41 | hypothetical protein PAK_P100075 [Pseudomonas phage PAK_P1] | hypothetical protein CDS | [YP_008869177.1](http://www.ncbi.nlm.nih.gov/protein/563448559?report=genbank&log$=prottop&blast_rank=1&RID=5VVPXKCM014) | 5.00E-18 | |
| EPA1_0127 | → | 72,030 | 72,995 | 966 | 4.64 kDa | pH 10.84 | thymidylate synthase [Pseudomonas phage vB_PaeM_C2-10_Ab1] | thymidylate synthase | [YP_007236936.1](http://www.ncbi.nlm.nih.gov/protein/431810048?report=genbank&log$=prottop&blast_rank=1&RID=5VVPXKCM014) | 0.0 | |
| EPA1_0128 | → | 72,997 | 73,341 | 345 | 36.84 kDa | pH 7.40 | hypothetical protein PaP1_gp111 [Pseudomonas phage PaP1] | hypothetical protein CDS | [YP_007236522.1](http://www.ncbi.nlm.nih.gov/protein/431809629?report=genbank&log$=prottop&blast_rank=1&RID=5VVPXKCM014) | 2.00E-79 | |
| EPA1_0129 | → | 73,358 | 74,404 | 1,047 | 13.19 kDa | pH 5.20 | ribonucleotide-diphosphate reductase beta subunit [Pseudomonas phage vB_PaeM_C2-10_Ab1] | ribonucleotide-diphosphate reductase beta subunit | [YP_007236938.1](http://www.ncbi.nlm.nih.gov/protein/431810050?report=genbank&log$=prottop&blast_rank=1&RID=5VVPXKCM014) | 0.0 | |
| EPA1_0130 | → | 74,397 | 76,142 | 1,746 | 13.19 kDa | pH 5.20 | putative ribonucleoside-diphosphate reductase alpha chain [Pseudomonas phage PAK_P4] | ribonucleoside-diphosphate reductase alphachain | [YP_008859290.1](http://www.ncbi.nlm.nih.gov/protein/563399449?report=genbank&log$=prottop&blast_rank=1&RID=5VVPXKCM014) | 0.0 | |
| EPA1_0131 | → | 76,217 | 76,363 | 147 | 40.37 kDa | pH 4.31 | hypothetical protein PAK_P200078 [Pseudomonas phage PAK_P2] | hypothetical protein CDS | [YP_008857119.1](http://www.ncbi.nlm.nih.gov/protein/563397354?report=genbank&log$=prottop&blast_rank=1&RID=5VVPXKCM014) | 1.00E-26 | |
| EPA1_0132 | → | 76,360 | 76,587 | 228 | 66.60 kDa | pH 6.72 | hypothetical protein PAK_P200079 [Pseudomonas phage PAK_P2] | hypothetical protein CDS | [YP_008857120.1](http://www.ncbi.nlm.nih.gov/protein/563397355?report=genbank&log$=prottop&blast_rank=1&RID=5VVPXKCM014) | 4.00E-46 | |
| EPA1_0133 | → | 76,587 | 76,820 | 234 | 5.61 kDa | pH 8.50 | hypothetical protein PJG4_135 [Pseudomonas phage JG004] | hypothetical protein CDS | [YP_007002405.1](http://www.ncbi.nlm.nih.gov/protein/418488350?report=genbank&log$=prottop&blast_rank=1&RID=5VVPXKCM014) | 5.00E-50 | |
| EPA1_0134 | → | 76,820 | 77,056 | 237 | 8.94 kDa | pH 8.20 | hypothetical protein PJG4_136 [Pseudomonas phage JG004] | hypothetical protein CDS | [YP_007002404.1](http://www.ncbi.nlm.nih.gov/protein/418488351?report=genbank&log$=prottop&blast_rank=1&RID=5VVPXKCM014) | 1.00E-48 | |
| EPA1_0135 | → | 77,056 | 77,370 | 315 | 8.89 kDa | pH 4.35 | hypothetical protein PJG4_137 [Pseudomonas phage JG004] | hypothetical protein CDS | [YP_007002403.1](http://www.ncbi.nlm.nih.gov/protein/418488352?report=genbank&log$=prottop&blast_rank=1&RID=5VVPXKCM014) | 1.00E-69 | |
| EPA1_0136 | → | 77,360 | 77,545 | 186 | 8.83 kDa | pH 5.57 | hypothetical protein BN405_2-10_Ab1_orf_123 [Pseudomonas phage vB_PaeM_C2-10_Ab1] | hypothetical protein CDS | [YP_007236944.1](http://www.ncbi.nlm.nih.gov/protein/431810056?report=genbank&log$=prottop&blast_rank=1&RID=5VVPXKCM014) | 2.00E-36 | |
| EPA1_0137 | → | 77,586 | 77,834 | 249 | 11.85 kDa | pH 4.54 | hypothetical protein PAK_P400085 [Pseudomonas phage PAK_P4] | hypothetical protein CDS | [YP_008859297.1](http://www.ncbi.nlm.nih.gov/protein/563399456?report=genbank&log$=prottop&blast_rank=1&RID=5VVPXKCM014) | 2.00E-51 | |
| EPA1_0138 | → | 77,846 | 78,331 | 486 | 6.90 kDa | pH 6.34 | hypothetical protein [Pseudomonas phage vB_PaeM_C2-10_Ab02] | hypothetical protein CDS | [CEF89066.1](http://www.ncbi.nlm.nih.gov/protein/713662224?report=genbank&log$=prottop&blast_rank=1&RID=5VVPXKCM014) | 5.00E-113 | |
| EPA1_0139 | → | 78,341 | 78,535 | 195 | 9.32 kDa | pH 4.49 | hypothetical protein PAK_P100089 [Pseudomonas phage PAK_P1] | hypothetical protein CDS | [YP_004327273.1](http://www.ncbi.nlm.nih.gov/protein/326804469?report=genbank&log$=prottop&blast_rank=1&RID=5VVPXKCM014) | 3.00E-37 | |
| EPA1_0140 | → | 78,537 | 78,767 | 231 | 17.40 kDa | pH 7.25 | hypothetical protein PAK_P100090 [Pseudomonas phage PAK_P1] | hypothetical protein CDS | [YP_004327274.1](http://www.ncbi.nlm.nih.gov/protein/326804470?report=genbank&log$=prottop&blast_rank=1&RID=5VVPXKCM014) | 3.00E-43 | |
| EPA1_0141 | → | 78,840 | 79,040 | 201 | 7.27 kDa | pH 9.58 | hypothetical protein PJG4_142 [Pseudomonas phage JG004] | hypothetical protein CDS | [YP_007002398.1](http://www.ncbi.nlm.nih.gov/protein/418488357?report=genbank&log$=prottop&blast_rank=1&RID=5VVPXKCM014) | 2.00E-39 | |
| EPA1_0142 | → | 79,207 | 80,193 | 987 | 8.69 kDa | pH 10.66 | hypothetical protein C11_138 [Pseudomonas phage C11] | hypothetical protein CDS | [ALJ97598.1](http://www.ncbi.nlm.nih.gov/protein/940162706?report=genbank&log$=prottop&blast_rank=1&RID=5VVPXKCM014) | 0.0 | |
| EPA1_0143 | → | 80,416 | 80,589 | 174 | 8.26 kDa | pH 10.10 | hypothetical protein PAK_P100093 [Pseudomonas phage PAK_P1] | hypothetical protein CDS | [YP_008869183.1](http://www.ncbi.nlm.nih.gov/protein/563448565?report=genbank&log$=prottop&blast_rank=1&RID=5VVPXKCM014) | 6.00E-32 | |
| EPA1_0144 | → | 81,218 | 81,487 | 270 | 6.89 kDa | pH 5.81 | hypothetical protein [Pseudomonas phage vB_PaeM_PAO1_Ab17] | hypothetical protein CDS | [CEF89620.1](http://www.ncbi.nlm.nih.gov/protein/713662824?report=genbank&log$=prottop&blast_rank=1&RID=5VVPXKCM014) | 2.00E-42 | |
| EPA1_0145 | → | 81,558 | 81,686 | 129 | 5.92 kDa | pH 7.84 | hypothetical protein PAK_P100099 [Pseudomonas phage PAK_P1] | hypothetical protein CDS | [YP_004327282.1](http://www.ncbi.nlm.nih.gov/protein/326804478?report=genbank&log$=prottop&blast_rank=1&RID=5VVPXKCM014) | 1.00E-18 | |
| EPA1_0146 | → | 81,686 | 81,994 | 309 | 9.93 kDa | pH 9.13 | hypothetical protein PAK_P200097 [Pseudomonas phage PAK_P2] | hypothetical protein CDS | [YP_008857138.1](http://www.ncbi.nlm.nih.gov/protein/563397373?report=genbank&log$=prottop&blast_rank=1&RID=5VVPXKCM014) | 5.00E-68 | |
| EPA1_0147 | → | 82,065 | 82,277 | 213 | 4.85 kDa | pH 11.82 | hypothetical protein Henu5_gp37 [Pseudomonas phage Henu5] | hypothetical protein CDS | [QAU05068.1](https://www.ncbi.nlm.nih.gov/protein/QAU05068.1?report=genbank&log$=prottop&blast_rank=1&RID=C0XD7Y8E014) | 4.00E-43 | |
| EPA1_0148 | → | 82,324 | 82,470 | 147 | 11.41 kDa | pH 10.25 | hypothetical protein PAK_P100101 [Pseudomonas phage PAK_P1] | hypothetical protein CDS | [YP_004327284.1](http://www.ncbi.nlm.nih.gov/protein/326804480?report=genbank&log$=prottop&blast_rank=1&RID=5VVPXKCM014) | 1.00E-21 | |
| EPA1_0148A | → | 82,565 | 82,714 | 150 | 6.89 kDa | pH 7.84 | hypothetical protein PAK_P200099 [Pseudomonas phage PAK_P2] | hypothetical protein CDS | [YP_008857140.1](http://www.ncbi.nlm.nih.gov/protein/563397375?report=genbank&log$=prottop&blast_rank=1&RID=HYY3B0UM014) | 9.00E-28 | |
| EPA1_0149 | → | 82,737 | 83,090 | 354 | 7.66 kDa | pH 10.67 | hypothetical protein PAK_P200100 [Pseudomonas phage PAK_P2] | hypothetical protein CDS | [YP_008857141.1](http://www.ncbi.nlm.nih.gov/protein/563397376?report=genbank&log$=prottop&blast_rank=1&RID=5VVPXKCM014) | 2.00E-78 | |
| EPA1_0150 | → | 83,166 | 83,837 | 672 | 5.66 kDa | pH 10.94 | hypothetical protein PJG4_154 [Pseudomonas phage JG004] | hypothetical protein CDS | [YP_007003117.1](http://www.ncbi.nlm.nih.gov/protein/418488369?report=genbank&log$=prottop&blast_rank=2&RID=5VVPXKCM014) | 1.00E-162 | |
| EPA1_0151 | → | 83,842 | 84,180 | 339 | 13.80 kDa | pH 8.89 | hypothetical protein PAK_P200103 [Pseudomonas phage PAK_P2] | hypothetical protein CDS | [YP_008857144.1](http://www.ncbi.nlm.nih.gov/protein/563397379?report=genbank&log$=prottop&blast_rank=1&RID=5VVS7VFA015) | 1.00E-78 | |
| EPA1_0152 | → | 84,180 | 84,533 | 354 | 24.32 kDa | pH 9.28 | hypothetical protein PJG4_156 [Pseudomonas phage JG004] | hypothetical protein CDS | [YP_007003118.1](http://www.ncbi.nlm.nih.gov/protein/418488371?report=genbank&log$=prottop&blast_rank=1&RID=5VVS7VFA015) | 2.00E-78 | |
| EPA1_0153 | → | 84,611 | 84,724 | 114 | 12.58 kDa | pH 9.45 | hypothetical protein PAK_P400103 [Pseudomonas phage PAK_P4] | hypothetical protein CDS | [YP_008859315.1](http://www.ncbi.nlm.nih.gov/protein/563399474?report=genbank&log$=prottop&blast_rank=1&RID=5VVS7VFA015) | 1.00E-18 | |
| EPA1_0154 | → | 84,797 | 84,991 | 195 | 13.02 kDa | pH 9.63 | hypothetical protein PAK_P100109 [Pseudomonas phage PAK_P1] | hypothetical protein CDS | [YP_008869192.1](http://www.ncbi.nlm.nih.gov/protein/563448574?report=genbank&log$=prottop&blast_rank=1&RID=5VVS7VFA015) | 1.00E-37 | |
| EPA1_0155 | → | 85,007 | 85,303 | 297 | 4.31 kDa | pH 10.48 | hypothetical protein PJG4_159 [Pseudomonas phage JG004] | hypothetical protein CDS | [YP_007002539.1](http://www.ncbi.nlm.nih.gov/protein/418488374?report=genbank&log$=prottop&blast_rank=1&RID=5VVS7VFA015) | 3.00E-62 | |
| EPA1_0156 | → | 85,300 | 85,524 | 225 | 7.03 kDa | pH 4.35 | hypothetical protein PAK_P200108 [Pseudomonas phage PAK_P2] | hypothetical protein CDS | [YP_008857149.1](http://www.ncbi.nlm.nih.gov/protein/563397384?report=genbank&log$=prottop&blast_rank=1&RID=5VVS7VFA015) | 4.00E-45 | |
| EPA1_0157 | → | 85,557 | 85,823 | 267 | 11.16 kDa | pH 4.34 | hypothetical protein PAK_P100112 [Pseudomonas phage PAK_P1] | hypothetical protein CDS | [YP_004327292.1](http://www.ncbi.nlm.nih.gov/protein/326804488?report=genbank&log$=prottop&blast_rank=1&RID=5VVS7VFA015) | 4.00E-56 | |
| EPA1_0158 | → | 85,820 | 86,212 | 393 | 8.34 kDa | pH 8.49 | hypothetical protein PAK_P400109 [Pseudomonas phage PAK_P4] | hypothetical protein CDS | [YP_008859321.1](http://www.ncbi.nlm.nih.gov/protein/563399480?report=genbank&log$=prottop&blast_rank=1&RID=5VVS7VFA015) | 2.00E-88 | |
| EPA1_0159 | → | 86,394 | 86,639 | 246 | 9.92 kDa | pH 3.81 | hypothetical protein PJG4_165 [Pseudomonas phage JG004] | hypothetical protein CDS | [YP_007003120.1](http://www.ncbi.nlm.nih.gov/protein/418488380?report=genbank&log$=prottop&blast_rank=1&RID=5VVS7VFA015) | 3.00E-49 | |
| EPA1_0160 | → | 86,819 | 86,965 | 147 | 15.44 kDa | pH 7.51 | hypothetical protein BN405_2-10_Ab1_orf_148 [Pseudomonas phage vB_PaeM_C2-10_Ab1] | hypothetical protein CDS | [YP_007236969.1](http://www.ncbi.nlm.nih.gov/protein/431810081?report=genbank&log$=prottop&blast_rank=1&RID=5VVS7VFA015) | 4.00E-22 | |
| EPA1_0161 | → | 87,001 | 87,483 | 483 | 9.25 kDa | pH 7.73 | hypothetical protein K8_168 [Pseudomonas phage K8] | hypothetical protein CDS | [ALF51489.1](http://www.ncbi.nlm.nih.gov/protein/930349042?report=genbank&log$=prottop&blast_rank=1&RID=5VVS7VFA015) | 2.00E-111 | |
| EPA1_0162 | → | 87,567 | 88,082 | 516 | 5.84 kDa | pH 3.81 | hypothetical protein K8_169 [Pseudomonas phage K8] | hypothetical protein CDS | [ALF51490.1](http://www.ncbi.nlm.nih.gov/protein/930349043?report=genbank&log$=prottop&blast_rank=1&RID=5VVS7VFA015) | 1.00E-117 | |
| EPA1_0163 | → | 88,198 | 88,410 | 213 | 19.45 kDa | pH 5.11 | hypothetical protein PAK_P30102 [Pseudomonas phage PAK_P3] | hypothetical protein CDS | [YP_008857736.1](http://www.ncbi.nlm.nih.gov/protein/563397974?report=genbank&log$=prottop&blast_rank=1&RID=5VVS7VFA015) | 1.00E-41 | |
| EPA1_0164 | → | 88,494 | 89,009 | 516 | 4.33 kDa | pH 10.49 | hypothetical protein PAK_P400114 [Pseudomonas phage PAK_P4] | hypothetical protein CDS | [YP_008859326.1](http://www.ncbi.nlm.nih.gov/protein/563399485?report=genbank&log$=prottop&blast_rank=1&RID=5VVS7VFA015) | 7.00E-113 | |
| EPA1_0165 | → | 89,080 | 89,313 | 234 | 7.92 kDa | pH 7.61 | hypothetical protein PAK_P200116 [Pseudomonas phage PAK_P2] | hypothetical protein CDS | [YP_008857157.1](http://www.ncbi.nlm.nih.gov/protein/563397392?report=genbank&log$=prottop&blast_rank=1&RID=5VVS7VFA015) | 3.00E-46 | |
| EPA1_0166 | → | 89,338 | 89,508 | 171 | 19.26 kDa | pH 9.25 | hypothetical protein [Pseudomonas phage vB_PaeM_PAO1_Ab03] | hypothetical protein CDS | [YP_009124549.1](http://www.ncbi.nlm.nih.gov/protein/764159843?report=genbank&log$=prottop&blast_rank=1&RID=5VVS7VFA015) | 1.00E-24 | |
| EPA1_0167 | → | 89,564 | 89,998 | 435 | 8.26 kDa | pH 5.55 | hypothetical protein BN405_2-10_Ab1_orf_153 [Pseudomonas phage vB_PaeM_C2-10_Ab1] | hypothetical protein CDS | [YP_007236974.1](http://www.ncbi.nlm.nih.gov/protein/431810086?report=genbank&log$=prottop&blast_rank=1&RID=5VVS7VFA015) | 5.00E-90 | |
| EPA1_0168 | → | 90,016 | 90,306 | 291 | 6.46 kDa | pH 11.32 | hypothetical protein K8_175 [Pseudomonas phage K8] | hypothetical protein CDS | [ALF51496.1](http://www.ncbi.nlm.nih.gov/protein/930349049?report=genbank&log$=prottop&blast_rank=1&RID=5VVS7VFA015) | 2.00E-59 | |
| EPA1_0160 | → | 90,390 | 90,584 | 195 | 16.57 kDa | pH 4.77 | hypothetical protein C11_169 [Pseudomonas phage C11] | hypothetical protein CDS | [ALJ97629.1](http://www.ncbi.nlm.nih.gov/protein/940162737?report=genbank&log$=prottop&blast_rank=1&RID=5VVS7VFA015) | 1.00E-37 | |
| EPA1_0170 | → | 90,768 | 90,968 | 201 | 11.44 kDa | pH 3.69 | hypothetical protein [Pseudomonas aeruginosa] | hypothetical protein CDS | [WP_058016447.1](http://www.ncbi.nlm.nih.gov/protein/953540921?report=genbank&log$=prottop&blast_rank=1&RID=HYY3U9CR015) | 6.00E-35 | |
