# Supplementary figures and images for: Synergistic Action of Phage and Antibiotics: Parameters to Enhance the Killing Efficacy Against Mono and Dual-Species Biofilms
Source: Antibiotics (Basel). 2019 Jul 25;8(3):103. doi: 10.3390/antibiotics8030103 (PMC6783858; doi:10.3390/antibiotics8030103)

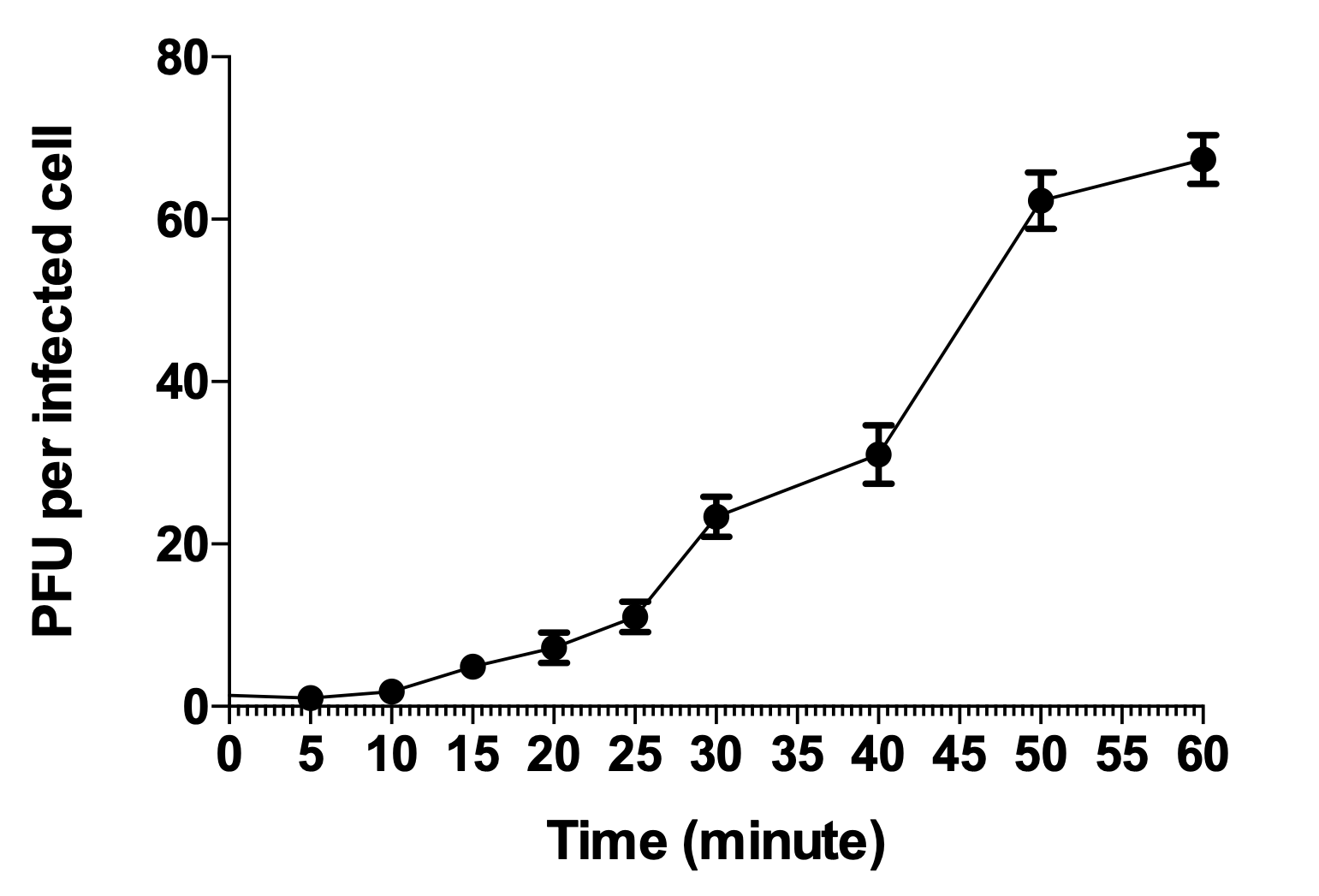

Supplement: Supplementary file 1 [file antibiotics-08-00103-s001.zip › Supplementary File/Figure S1.png]

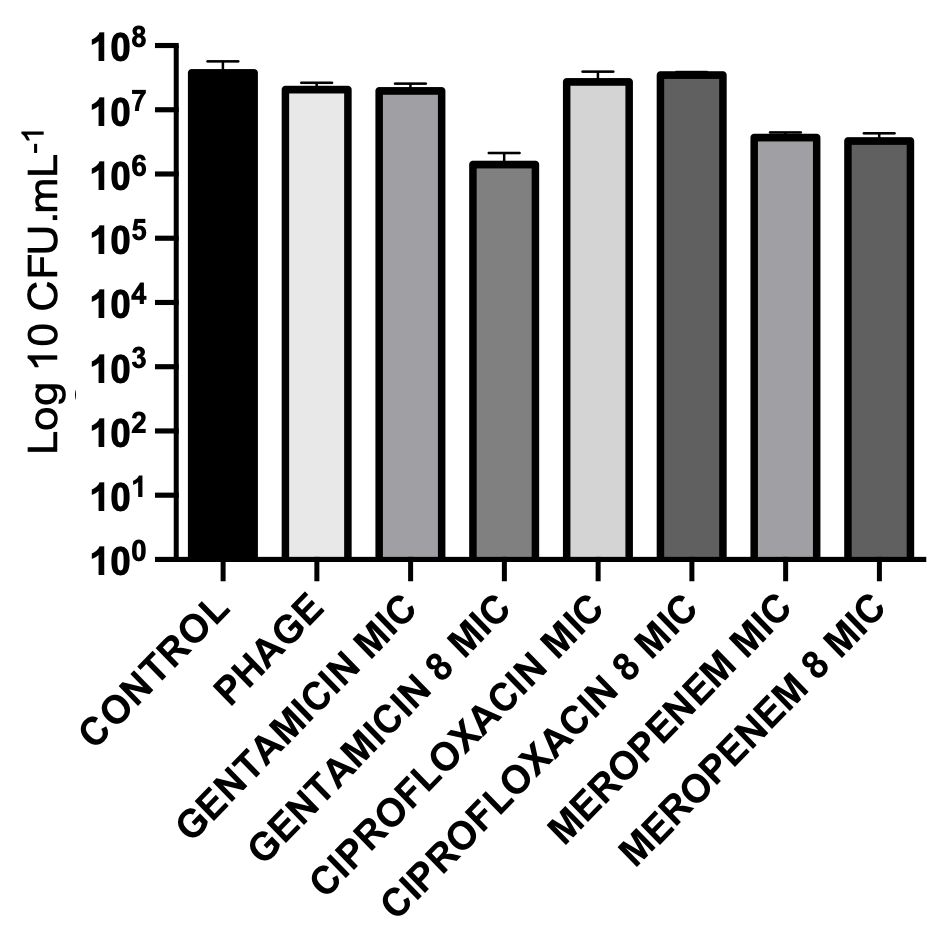

Supplement: Supplementary file 1 [file antibiotics-08-00103-s001.zip › Supplementary File/Figure S2.png]

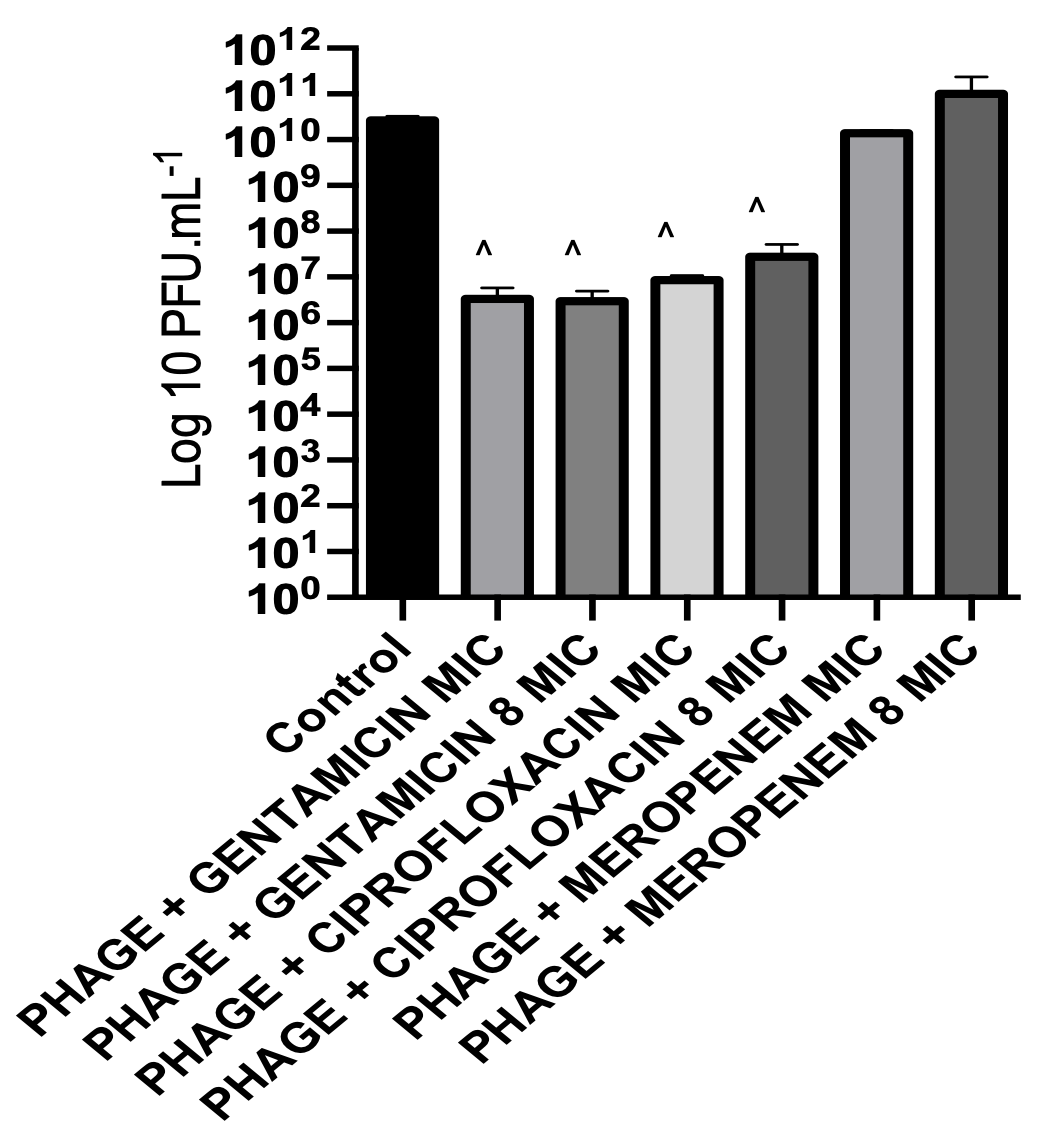

Supplement: Supplementary file 1 [file antibiotics-08-00103-s001.zip › Supplementary File/Figure S3.png]

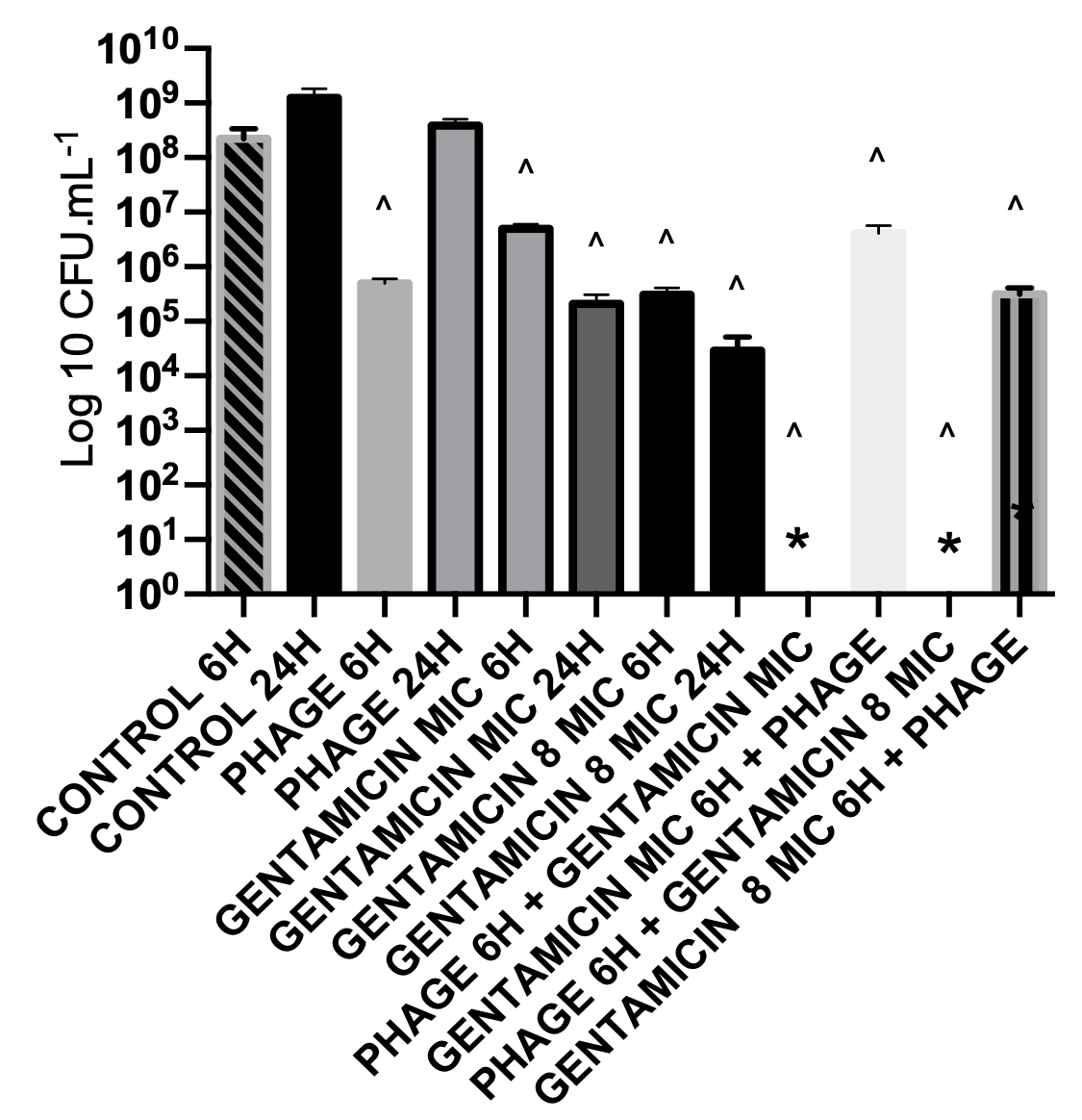

Supplement: Supplementary file 1 [file antibiotics-08-00103-s001.zip › Supplementary File/Figure S4.png]
